# Identity-Based Matchmaking Encryption with Equality Test

**DOI:** 10.3390/e26010074

**Published:** 2024-01-15

**Authors:** Zhen Yan, Xijun Lin, Xiaoshuai Zhang, Jianliang Xu, Haipeng Qu

**Affiliations:** College of Computer Science and Technology, Ocean University of China, Qingdao 266100, Chinalinxj77@ouc.edu.cn (X.L.); xjl9898@ouc.edu.cn (J.X.)

**Keywords:** matchmaking encryption, equality test, confidentiality, authenticity, anonymity

## Abstract

The identity-based encryption with equality test (IBEET) has become a hot research topic in cloud computing as it provides an equality test for ciphertexts generated under different identities while preserving the confidentiality. Subsequently, for the sake of the confidentiality and authenticity of the data, the identity-based signcryption with equality test (IBSC-ET) has been put forward. Nevertheless, the existing schemes do not consider the anonymity of the sender and the receiver, which leads to the potential leakage of sensitive personal information. How to ensure confidentiality, authenticity, and anonymity in the IBEET setting remains a significant challenge. In this paper, we put forward the concept of the identity-based matchmaking encryption with equality test (IBME-ET) to address this issue. We formalized the system model, the definition, and the security models of the IBME-ET and, then, put forward a concrete scheme. Furthermore, our scheme was confirmed to be secure and practical by proving its security and evaluating its performance.

## 1. Introduction

The swift progress in cloud computing featured by the outsourcing of data to the cloud has given rise to a growing trend among organizations and individuals, enabling entities to benefit from the ultra-large capacity and calculating services provided by cloud providers. The maintenance of data confidentiality is a fundamental security requirement of cloud storage, which is generally achieved by employing existing cryptographic mechanisms. Nonetheless, how to perform efficient searches on ciphertexts is a practical problem. In order to protect data confidentiality and, meanwhile, support privacy-preserving keyword searching on ciphertexts, public key encryption with keyword search (PEKS) has been presented [1]. Nevertheless, PEKS is limited to searching on ciphertexts generated under a single public key, rendering it unsuitable for cloud storage scenarios involving multiple users.

To provide privacy-preserving equality searching on ciphertexts encrypted under distinct public keys without losing the data confidentiality, Yang et al. [2] put forward an extension of PEKS known as the public key encryption with equality test (PKEET). However, in Yang et al.’s construction, anyone can conduct the equality test without authorization, which infringes on the data owner’s privacy. Hence, the authorization mechanism was introduced into the PKEET to guarantee that no one except the data owner can enable the cloud server to test its ciphertexts with the others’.

Subsequently, Ma [3] proposed the identity-based encryption with equality test (IBEET) to eliminate the certificate management problem of the PKEET. In this primitive, the identities of the sender and receiver were exploited to denote the public keys, eliminating the need for certificate management. Owing to the equality test function, the IBEET has been applied in various practical applications, such as personal health record (PHR) systems [4,5] and Internet of Vehicles (IoV) road monitoring [6].

Ensuring the authenticity of data is another fundamental security requirement of cloud storage. For the sake of the confidentiality and authenticity of data while supporting the privacy-preserving equality test for ciphertexts generated from different identities, Xiong et al. [7] presented the identity-based signcryption with equality test (IBSC-ET). Afterwards, several related signcryption schemes supporting the equality test have been conceived of. Nevertheless, the existing studies have not considered the anonymity of the sender and the receiver, which leads to the potential leakage of sensitive personal information.

### 1.1. Motivation

**Figure 1:**
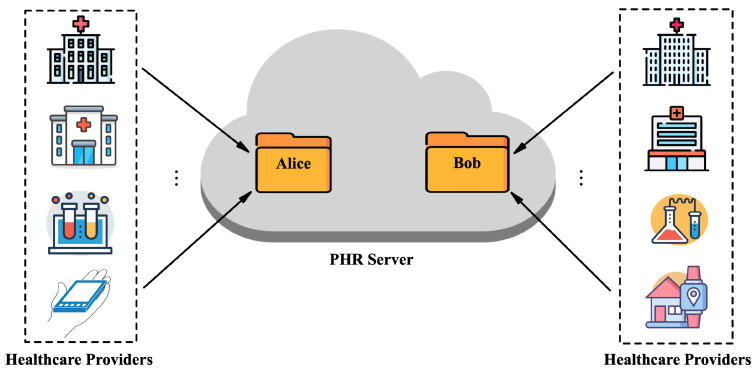
PHR system model.

As depicted in Figure 1, in a PHR system, the patients’ PHRs contain as much relevant health data as possible from various healthcare providers over their lifetime. To ensure patients’ privacy, it is essential to store the health data in the cloud in ciphertext form. To find patients having similar illnesses, a patient (e.g., Alice or Bob) can authorize the cloud server to compare his/her ciphertexts sent by a specified healthcare provider with the others’ ciphertexts, so that the patients can help each other by sharing their experiences or mental processes.

However, by employing the existing signcryption schemes with equality test (to guarantee the confidentiality and authenticity of health data while supporting the privacy-preserving equality test on ciphertexts), the patients are unable to prevent sensitive personal information from being leaked to the cloud server. That is because the existing schemes do not consider the anonymity of the sender and receiver of the ciphertext. Consequently, the cloud server can know the healthcare provider of the ciphertext, e.g., MD Anderson Cancer Center. Likewise, from the ciphertext and the authorization trapdoor, the cloud server can learn whose identity the ciphertext is encrypted under, namely who is the receiver of the ciphertext, in this way to identify the patient associated with the ciphertext. Obviously, this seriously infringes upon the patient’s privacy.

Hence, during the equality testing procedure, there are three security aspects that should be guaranteed against the cloud server:

1. **Confidentiality:** The cloud server has no knowledge about the health data concealed in the ciphertext.
2. **Authenticity:** The cloud server is unable to fake any legitimate ciphertext pertaining to the sender and the receiver.
3. **Anonymity:** The cloud server has no knowledge about the identities of the sender and the receiver concealed in the ciphertext.

Therefore, we propose a new primitive, which not only offers the confidentiality, authenticity, and anonymity of data stored in the cloud, but also provides equality test functionality for ciphertexts generated under different identities without losing the confidentiality, authenticity, and anonymity of the data.

### 1.2. Related Works

**Search on ciphertexts:** Searchable encryption (SE) [8] was put forward to offer secure search functionality over ciphertexts encrypted under single public key. There are two categories of SE: public key encryption with keyword search (PEKS) [1,9,10] and symmetric searchable encryption (SSE) [11,12]. PEKS was conceived of by Boneh et al. [1] to support keyword searching over ciphertexts in public key settings by using the corresponding trapdoors without retrieving messages. After that, a variety of PEKS schemes have been presented for enhanced functionalities and different application requirements [9,10]. However, SE cannot offer equality test functionality for ciphertexts generated under different identities, which differs from our proposal.

**Equality test on ciphertexts:** The primitive of the PKEET was put forward to verify whether the identical message is concealed in two ciphertexts, where the ciphertexts may be encrypted under distinct public keys [2]. Then, the authorization mechanisms were introduced into the PKEET, and a series of PKEET schemes supporting various authorizations were proposed [13,14]. Ma [3] first introduced the primitive of the IBEET, to eliminate the certificate management problem of the traditional PKEET. A semi-generic IBEET scheme was conceived of by Lee et al. [15] to achieve CCA security. Then, several IBEET schemes supporting various authorizations were introduced [16,17]. Although the above schemes offer equality test functionality while preserving the confidentiality, the data authenticity is not guaranteed. To address this challenge, Xiong et al. [7] established the notion of the IBSC-ET by combining identity-based signcryption (IBSC) [18] and the IBEET. Afterwards, several signcryption schemes with equality test functionality for heterogeneous systems were proposed [19,20,21]. However, the existing studies have not considered the anonymity of the sender and the receiver, which leads to the potential leakage of sensitive personal information, which differs from our proposal.

**Identity-based matchmaking encryption:** In CRYPTO 2019, Ateniese et al. [22] put forward the primitive of identity-based matching encryption (IB-ME) to logically ensure the confidentiality, authenticity, and anonymity of data in one step. The guarantee of IB-ME is as follows: the recipient obtains the message when the match happens (both parties’ identities match the identity specified by the other party); in case the match does not happen, no information is disclosed other than the fact of the mismatch. Then, by extending IB-ME, a secure access control scheme was conceived of by Xu et al. [23] for cloud–fog computing, and a secure access control scheme was suggested by Sun et al. [24] for cloud-enabled industrial IoT healthcare systems. Chen et al. [25] suggested an IB-ME scheme on the basis of standard assumptions. Wu et al. [26] conceived of a Fuzzy IB-ME scheme. Yan et al. [27] conceived of an IB-ME scheme supporting proxy decryption. Sun et al. [28] suggested an IB-ME scheme supporting a broadcast mechanism. However, although IB-ME can ensure the confidentiality, authenticity, and anonymity of data, all of these related schemes cannot offer equality test functionality for ciphertexts without losing the confidentiality, authenticity, and anonymity of the data, which differs from our proposal.

### 1.3. Contributions

We emphasize here again that the existing cryptographic schemes with the equality test do not consider the anonymity of the sender and the receiver, which leads to the potential leakage problem of sensitive personal information. Hence, we put forward a novel primitive, called the identity-based matchmaking encryption with equality test (IBME-ET), by combining IB-ME and the IBEET. This primitive not only offers the confidentiality, authenticity, and anonymity of data stored in the cloud, but also provides equality test functionality for ciphertexts generated under different identities without losing the confidentiality, authenticity, and anonymity of the data.

Our proposed IBME-ET can advance the anonymity of existing applications. For example, in a PHR system [4,5], the patient can permit the cloud server to compare his/her encrypted health data sent by a specified healthcare provider with the others’, in this way to make friends with the patients having a similar illness. Our proposal can simplify the leakage problem of the real identities of the healthcare provider and the patient, which exists in current cryptographic schemes with the equality test, thereby guaranteeing the confidentiality, authenticity, and anonymity of the patients’ health data.

The equality testing process in the IBME-ET can be succinctly outlined as follows: Let $C_{\left(\sigma_{A},{rcv}_{A}\right)}$ denote a ciphertext generated on $\left(ek_{\sigma_{A}},{rcv}_{A},m_{A}\right)$ and $C_{\left(\sigma_{B},{rcv}_{B}\right)}$ denote a ciphertext generated on $\left(ek_{\sigma_{B}},{rcv}_{B},m_{B}\right)$, where $ek_{\sigma_{A}}$ and $ek_{\sigma_{B}}$ are the encryption keys of the senders with identities $\sigma_{A}$ and $\sigma_{B}$ and $rcv_{A}$ and $rcv_{B}$ are the identities of the specified receivers, respectively. Furthermore, let $td_{\left(snd_{A},\rho_{A}\right)}$ be a trapdoor generated on $\left(snd_{A},dk_{\rho_{A}}\right)$ and $td_{\left(snd_{B},\rho_{B}\right)}$ be a trapdoor generated on $\left(snd_{B},dk_{\rho_{B}}\right)$, where $snd_{A}$ and $snd_{B}$ are the identities of the specified senders and $dk_{\rho_{A}}$ and $dk_{\rho_{B}}$ are the decryption keys of the receivers with identities $\rho_{A}$ and $\rho_{B}$, respectively. Given $\left(C_{\left(\sigma_{A},{rcv}_{A}\right)},td_{\left(snd_{A},\rho_{A}\right)}\right)$ and $\left(C_{\left(\sigma_{B},{rcv}_{B}\right)},td_{\left(snd_{B},\rho_{B}\right)}\right)$, two conditions are involved:

- Match (i.e., $\sigma_{A}={snd}_{A}\;\wedge \;{rcv}_{A}=\rho_{A}\;\wedge \;\sigma_{B}={snd}_{B}\;\wedge \;{rcv}_{B}=\rho_{B}\;\wedge m_{A}=m_{B}$): the cloud server returns 1, and no further information is revealed other than the fact that the match happened, that is the cloud server learns neither the messages $m_{A}=m_{B}$ nor the identities $\sigma_{A}={snd}_{A}$, ${rcv}_{A}=\rho_{A}$, $\sigma_{B}={snd}_{B}$, ${rcv}_{B}=\rho_{B}$.
- Mismatch (i.e., $\sigma_{A}\neq {snd}_{A}\;\vee \;{rcv}_{A}\neq \rho_{A}\;\vee \;\sigma_{B}\neq {snd}_{B}\;\vee \;{rcv}_{B}\neq \rho_{B}\;\vee m_{A}\neq m_{B}$): the cloud server returns 0, and no further information is revealed other than the fact of the mismatch, that is the cloud server learns neither the messages $m_{A}$, $m_{B}$ nor the identities $\sigma_{A}$, ${snd}_{A}$, ${rcv}_{A}$, $\rho_{A}$, $\sigma_{B}$, ${snd}_{B}$, ${rcv}_{B}$, $\rho_{B}$.

The principal contributions can be succinctly outlined as follows:

1. We present the notion of the IBME-ET, which not only offers the confidentiality, authenticity, and anonymity of data stored in the cloud, but also provides equality test functionality for ciphertexts generated under different identities without losing the confidentiality, authenticity, and anonymity of the data.
2. We put forward the system model and definition of the IBME-ET. With respect to the confidentiality, authenticity, and anonymity, we formulated four security models for the IBME-ET by taking four types of adversaries into account.
3. We constructed a concrete IBME-ET scheme on the basis of the BDH assumption and the Gap-BDH assumption. Our scheme was confirmed to be secure and practical by proving its security and evaluating its performance.

### 1.4. Organization

In general: Section 2 introduces the preliminaries while Section 3 presents IBME-ET by displaying its system, definition and four security models. Section 4 and Section 5, respectively, focus on the detailed scheme and analysis of security. Then, Section 6 focuses on performance evaluation, Section 7 arrives at a conclusion.

## 2. Preliminaries

### 2.1. Asymmetric Bilinear Groups

$G,\hat{G}$, and $G_{T}$ indicate three multiplicative cyclic groups with prime order *q*. *g* and $\hat{g}$ are the generators of G and $\hat{G}$, respectively. An asymmetric bilinear map $e:G\times \hat{G}\to G_{T}$ includes the following characteristics:

1. *Bilinearity:*∀x∈G, $\forall y\in \hat{G}$ and $\forall u,v\in Z_{q}^{*}$, $e(x^{u},y^{v})=$$e{\left(x,y\right)}^{uv}$.
2. *Non-degeneracy:*∃g∈G, $\hat{g}\in \hat{G}$, $e(g,\hat{g})\neq 1$.

Note that the group operations and asymmetric bilinear map *e* can be computed efficiently. However, if no efficiently computable isomorphisms are found between G and $\hat{G}$, then $G,\hat{G}$ and $G_{T}$ do not possess efficiently computable isomorphisms.

### 2.2. Assumptions

1. *Bilinear Diffie–Hellman (BDH) assumption:* When a tuple $(g,g^{a},g^{c},\hat{g},$ ${\hat{g}}^{a},{\hat{g}}^{b})\in G^{3}\times {\hat{G}}^{3}$ is given, no PPT algorithm A calculates $e{\left(g,\hat{g}\right)}^{abc}\in G_{T}$ with non-negligible advantage. Define A’s advantage as
  $$Adv_{BDH}^{A}\left(\lambda \right)=\mathrm{Pr}\left[A\left(g,g^{a},g^{c},\hat{g},{\hat{g}}^{a},{\hat{g}}^{b}\right)=e{\left(g,\hat{g}\right)}^{abc}\right].$$
2. *Gap-bilinear Diffie–Hellman (Gap-BDH) assumption:* When a tuple $\left(g,g^{a},g^{c},\hat{g},{\hat{g}}^{a},{\hat{g}}^{b}\right)\in G^{3}\times {\hat{G}}^{3}$ is given, even with the decision BDH oracle $O_{\mathrm{DBDH}}$, no PPT algorithm A calculates $e{\left(g,\hat{g}\right)}^{abc}\in$$G_{T}$ with non-negligible advantage [29]. Tuples of the form $(g,g^{a},g^{c},$ $\hat{g},{\hat{g}}^{a},{\hat{g}}^{b},e{\left(g,\hat{g}\right)}^{abc})$ are known as “BDH tuples”. With $(g,g^{a},$$g^{c},\hat{g},{\hat{g}}^{a},{\hat{g}}^{b},T)$, $O_{\mathrm{DBDH}}$ is able to check $T=e{\left(g,\hat{g}\right)}^{abc}$ or not. $O_{\mathrm{DBDH}}$ outputs 1 when $T=e{\left(g,\hat{g}\right)}^{abc}$; otherwise, $O_{\mathrm{DBDH}}$ outputs 0. Define A’s advantage as
  $$Adv_{Gap-BDH}^{A}\left(\lambda \right)=\mathrm{Pr}\left[A\left(g,g^{a},g^{c},\hat{g},{\hat{g}}^{a},{\hat{g}}^{b},O_{\mathrm{DBDH}}\right)=e{\left(g,\hat{g}\right)}^{abc}\right].$$

## 3. Definitions of IBME-ET

### 3.1. System Model

**Figure 2:**
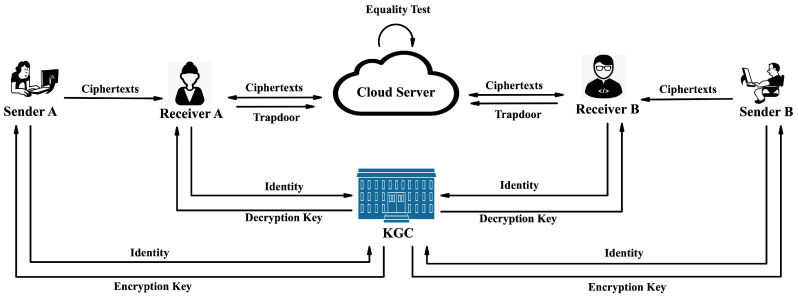
IBME-ET system model.

In Figure 2, our proposed IBME-ET comprises four distinct entities.

- *KGC:* This entity’s responsibility is to securely generate and distribute encryption keys and decryption keys.
- *Sender:* This entity’s responsibility is to generate ciphertexts, ensuring the confidentiality, authenticity, and anonymity of the data.
- *Receiver:* This entity is responsible for collecting and outsourcing ciphertexts from potential senders secretly. It permits the cloud server to test ciphertexts sent by a specific sender without compromising the confidentiality, authenticity, and anonymity of the data.
- *Cloud server:* This entity’s responsibility is to store the ciphertexts and perform equality tests based on the receivers’ authorizations.

Our workflow is succinctly outlined as follows:

1. The KGC utilizes the algorithm *SKGen* to calculate the encryption key $ek_{\sigma }$ in accordance with the identity of the sender σ and securely delivers this to the sender. Similarly, the KGC utilizes the algorithm *RKGen* to calculate the decryption key $dk_{\rho }$ in accordance with the identity of the receiver ρ and securely delivers this to the receiver.
2. A sender identified as σ executes the algorithm *Enc* to conceal the message *m* using encryption key $ek_{\sigma }$ along with a target receiver’s identity rcv, delivering it to the receiver with the ciphertext $C_{\left(\sigma ,rcv\right)}$.
3. A receiver identified as ρ executes the algorithm *Decc* to decrypt the ciphertexts by employing the receiver’s decryption key $dk_{\rho }$ and the identity of the target sender snd, delivering the desirable ciphertexts to the cloud server. Specifically, given $C_{\left(\sigma ,rcv\right)}$, $dk_{\rho }$, and snd, the guarantee in the decryption procedure is as follows:
  - Match (i.e., σ=snd∧ρ=rcv): the message *m* is obtained by the receiver.
  - Mismatch (i.e., σ≠snd∨ρ≠rcv): the receiver obtains neither the message *m* nor the identities σ, rcv.
4. To test the ciphertexts offered by a target sender, the receiver identified as ρ executes the algorithm *Auth* to calculate a trapdoor $td_{\left(snd,\rho \right)}$ with the identity of the target sender snd and its decryption key $dk_{\rho }$ and delivers the trapdoor to the cloud server.
5. Utilizing the receivers’ trapdoors, the cloud server executes the algorithm *Test* to test the ciphertexts sent by the specified senders without learning the messages and identities. Specifically, given $\left(C_{\left(\sigma_{A},{rcv}_{A}\right)},td_{\left(snd_{A},\rho_{A}\right)}\right)$ and $\left(C_{\left(\sigma_{B},{rcv}_{B}\right)},td_{\left(snd_{B},\rho_{B}\right)}\right)$, the guarantee in equality testing procedure is as follows:
  - Match (i.e., $\sigma_{A}={snd}_{A}\;\wedge \;{rcv}_{A}=\rho_{A}\;\wedge \;\sigma_{B}={snd}_{B}\;\wedge \;{rcv}_{B}=\rho_{B}\;\wedge m_{A}=m_{B}$): the cloud server returns 1, and the cloud server learns neither the messages $m_{A}=m_{B}$ nor the identities $\sigma_{A}={snd}_{A}$, ${rcv}_{A}=\rho_{A}$, $\sigma_{B}={snd}_{B}$, ${rcv}_{B}=\rho_{B}$.
  - Mismatch (i.e., $\sigma_{A}\neq {snd}_{A}\;\vee \;{rcv}_{A}\neq \rho_{A}\;\vee \;\sigma_{B}\neq {snd}_{B}\;\vee \;{rcv}_{B}\neq \rho_{B}\;\vee m_{A}\neq m_{B}$): the cloud server returns 0, and the cloud server learns neither the messages $m_{A}$, $m_{B}$ nor the identities $\sigma_{A}$, ${snd}_{A}$, ${rcv}_{A}$, $\rho_{A}$, $\sigma_{B}$, ${snd}_{B}$, ${rcv}_{B}$, $\rho_{B}$.

### 3.2. IBME-ET Definition

An IBME-ET scheme comprises the subsequent algorithms:

- Setup(λ)→(pp,mk): The system parameters pp along with the master key mk are answered.
- $SKGen\left(pp,mk,\sigma \right)\to ek_{\sigma }$: The encryption key $ek_{\sigma }$ for the sender identified as σ is answered.
- $RKGen\left(pp,mk,\rho \right)\to dk_{\rho }$: The decryption key $dk_{\rho }$ for the receiver identified as ρ is answered.
- $Enc(pp,ek_{\sigma },rcv,m)\to C$: Given the system parameters pp, an encryption key of the sender $ek_{\sigma }$, and an identity of the target receiver rcv along with the message *m*, the corresponding ciphertext *C* is answered.
- $Dec(pp,dk_{\rho },snd,C)\to m/⊥$: Given the system parameters pp, a decryption key of the receiver $dk_{\rho }$, and an identity of the target sender snd along with the ciphertext *C*, the corresponding message *m* is answered or the symbol ⊥ to signal the failure of the decryption is answered.
- $Auth\left(pp,snd,dk_{\rho }\right)\to td_{\left(snd,\rho \right)}$: Given the system parameters pp and an identity of the target sender snd along with a decryption key of the receiver $dk_{\rho }$, the corresponding trapdoor $td_{\left(snd,\rho \right)}$ is answered.
- $Test(pp,C_{\left(\sigma_{A},{rcv}_{A}\right)},td_{\left({snd}_{A},\rho_{A}\right)},C_{\left(\sigma_{B},{rcv}_{B}\right)},td_{\left({snd}_{B},\rho_{B}\right)})\to 0/1$: Given the system parameters pp, two pairs of ciphertext/trapdoors $\left(C_{\left(\sigma_{A},{rcv}_{A}\right)},td_{\left({snd}_{A},\rho_{A}\right)}\right)$ and $\left(C_{\left(\sigma_{B},{rcv}_{B}\right)},td_{\left({snd}_{B},\rho_{B}\right)}\right)$, if $\sigma_{A}={snd}_{A}\;\wedge \;{rcv}_{A}=\rho_{A}\;\wedge \;\sigma_{B}={snd}_{B}\;\wedge \;{rcv}_{B}=\rho_{B}\;\wedge \;C_{\left(\sigma_{A},{rcv}_{A}\right)}$ and $C_{\left(\sigma_{B},{rcv}_{B}\right)}$ are generated using the identical message, it answers 1. Otherwise, it answers 0.

*Correctness:* An IBME-ET scheme is correct when the subsequent conditions are met:

1. When σ=snd∧ρ=rcv, $Dec(pp,dk_{\rho },snd,Enc(pp,$ $ek_{\sigma },rcv,m))=m$ always holds.
2. Let $C_{\left(\sigma_{A},{rcv}_{A}\right)}=Enc\left(pp,ek_{\sigma_{A}},{rcv}_{A},m_{A}\right)$, $C_{\left(\sigma_{B},{rcv}_{B}\right)}=Enc\left(pp,ek_{\sigma_{B}},{rcv}_{B},m_{B}\right)$, $td_{\left(snd_{A},\rho_{A}\right)}=Auth\left(pp,snd_{A},dk_{\rho_{A}}\right)$, and $td_{\left(snd_{B},\rho_{B}\right)}=Auth\left(pp,snd_{B},dk_{\rho_{B}}\right)$. If $\sigma_{A}={snd}_{A}\;\wedge \;{rcv}_{A}=\rho_{A}$$\;\wedge \;\sigma_{B}={snd}_{B}\;\wedge \;{rcv}_{B}=\rho_{B}$$\;\wedge \;m_{A}=m_{B}$, $Test(pp,C_{\left(\sigma_{A},{rcv}_{A}\right)}$, $td_{\left(snd_{A},\rho_{A}\right)},C_{\left(\sigma_{B},{rcv}_{B}\right)},td_{\left(snd_{B},\rho_{B}\right)})=1;$ otherwise, $\mathrm{Pr}[Test(pp,C_{\left(\sigma_{A},{rcv}_{A}\right)},td_{\left(snd_{A},\rho_{A}\right)}$, $C_{\left(\sigma_{B},{rcv}_{B}\right)},td_{\left(snd_{B},\rho_{B}\right)})=1]$ is negligible.

### 3.3. Security Definitions

With respect to the confidentiality, authenticity, and anonymity of the IBME-ET, it is crucial to consider four distinct types of adversaries:

- *Type-I adversary $A_{1}$*: Without the trapdoor and decryption key of the receiver, $A_{1}$ is unable to determine which message the challenge ciphertext is computed from. For $A_{1}$, define the security model IND-ID-CCA.
- *Type-II adversary $A_{2}$*: Without the decryption key of the receiver, $A_{2}$ is unable to obtain the message concealed in the challenge ciphertext. For $A_{2}$, define the security model OW-ID-CCA.
- *Type-III adversary $A_{3}$*: Without the decryption key of the receiver and the encryption key of the sender, $A_{3}$ is unable to determine the corresponding sender and receiver, even if $A_{3}$ has the trapdoor. For $A_{3}$, define the security model ANON-ID-CCA.
- *Type-IV adversary $A_{4}$*: Without the decryption key of the receiver and the encryption key of the sender, $A_{4}$ is unable to fake any legitimate ciphertext delivered by the sender to the receiver, even if $A_{4}$ has the trapdoor. For $A_{4}$, define the security model sUF-ID-CMA.

Let C be the challenger. We have the following oracles:

- $O_{SKGen}\left(\sigma_{i}\right)$: Once the identity of the sender $\sigma_{i}$ is received, C answers the encryption key $ek_{\sigma_{i}}$.
- $O_{RKGen}\left(\rho_{j}\right)$: Once the identity of the receiver $\rho_{j}$ is received, C answers the decryption key $dk_{\rho_{j}}$.
- $O_{Enc}\left(\sigma_{i},rcv,m\right)$: Once the identity of the sender $\sigma_{i}$, the identity of the target receiver rcv, and a message *m* are received, C answers the result of $Enc(pp,ek_{\sigma_{i}},rcv,m)$.
- $O_{Dec}\left(\rho_{j},snd,C\right)$: Once the identity of the receiver $\rho_{j}$, the identity of the target sender snd, and a ciphertext *C* are received, C answers the result of $Dec(pp,dk_{\rho_{j}},snd,C)$.
- $O_{Auth}\left(snd,\rho_{j}\right)$: Once the identity of the target sender snd and the identity of the receiver $\rho_{j}$ are received, C answers the corresponding trapdoor $td_{\left(snd,\rho_{j}\right)}=Auth\left(pp,snd,dk_{\rho_{j}}\right)$.

**Definition** **1** (IND-ID-CCA)**.** *Regarding $A_{1}$, the IBME-ET scheme meets IND-ID-CCA security when no PPT $A_{1}$ is winning the game below with a non-negligible advantage:*

*1*. *Setup: C utilizes the algorithm Setup to calculate the master key mk and the system parameters pp and delivers pp to $A_{1}$.*
*2*. *Phase 1: $A_{1}$ can issue queries to the oracles: $O_{SKGen}$, $O_{RKGen}$, $O_{Auth}$, $O_{Dec}$.*
*3*. Challenge: $A_{1}$ sends identities $\sigma^{*},{rcv}^{*}$ and equal-length messages $m_{0}^{*},m_{1}^{*}$ to C. Subsequently, C randomly selects x∈{0,1} and answers $A_{1}$ with the challenge ciphertext $C^{*}=Enc\left(pp,ek_{\sigma^{*}},{rcv}^{*},m_{x}^{*}\right)$.
*4*. *Phase 2: $A_{1}$ makes queries like in Phase 1.*
*5*. *Guess: $A_{1}$ answers a guess $x^{\prime }\in \left\{0,1\right\}$ and is winning when $x=x^{\prime }$. $A_{1}$’s advantage is defined as $Adv_{IBME-ET,A_{1}}^{IND-ID-CCA}\left(\lambda \right)=|$Pr$\left[x=x^{\prime }\right]-\frac{1}{2}|.$*

In the above game, the constraint is that $A_{1}$ cannot ask the following queries: $O_{RKGen}\left({rcv}^{*}\right)$, $O_{Auth}\left(\sigma^{*},{rcv}^{*}\right)$, $O_{Dec}\left({rcv}^{*},\sigma^{*},C^{*}\right)$.

**Definition** **2** (OW-ID-CCA)**.** *Regarding $A_{2}$, the IBME-ET scheme meets OW-ID-CCA security when no PPT $A_{2}$ is winning the game below with a non-negligible advantage:*

*1*. *Setup: Same as Definition 1.*
*2*. *Phase 1: $A_{2}$ can issue queries to the oracles: $O_{SKGen}$, $O_{RKGen}$, $O_{Auth}$, $O_{Dec}$.*
*3*. Challenge: $A_{2}$ sends identities $\sigma^{*},{rcv}^{*}$ to C. Subsequently, C randomly chooses a message $m^{*}\in {\left\{0,1\right\}}^{\lambda }$ and answers to $A_{2}$ with the challenge ciphertext $C^{*}=Enc\left(pp,ek_{\sigma^{*}},{rcv}^{*},m^{*}\right)$.
*4*. *Phase 2: $A_{2}$ makes queries like in Phase 1.*
*5*. Guess: $A_{2}$ answers a guess $m^{\prime }$ and is winning when $m^{*}=m^{\prime }$. $A_{2}$’s advantage is defined as $Adv_{IBME-ET,A_{2}}^{OW-ID-CCA}\left(\lambda \right)=$Pr$[m^{*}=m^{\prime }].$

In the above game, the constraints is that $A_{2}$ cannot ask the following queries: $O_{RKGen}\left({rcv}^{*}\right)$, $O_{Dec}\left({rcv}^{*},\sigma^{*},C^{*}\right)$.

**Definition** **3** (ANON-ID-CCA)**.** *Regarding $A_{3}$, the IBME-ET scheme meets ANON-ID-CCA security when no PPT $A_{3}$ is winning the game below with a non-negligible advantage:*

*1*. *Setup: Same as Definition 1.*
*2*. *Phase 1: $A_{3}$ can issue queries to the oracles: $O_{SKGen}$, $O_{RKGen}$, $O_{Auth}$, $O_{Enc}$, $O_{Dec}$.*
*3*. Challenge: $A_{3}$ sends identities $\left({snd}_{0}^{*},\rho_{0}^{*}\right)$, $\left({snd}_{1}^{*},\rho_{1}^{*}\right)$ and a message $m^{*}$ to C. Subsequently, C randomly chooses x∈{0,1} and answers to $A_{3}$ with the challenge ciphertext $C^{*}=Enc\left(pp,ek_{{snd}_{x}^{*}},\rho_{x}^{*},m^{*}\right)$ and the challenge trapdoor $td_{\left({snd}_{x}^{*},\rho_{x}^{*}\right)}=Auth\left(pp,{snd}_{x}^{*},dk_{\rho_{x}^{*}}\right)$.
*4*. *Phase 2: $A_{3}$ makes queries like in Phase 1.*
*5*. *Guess: $A_{3}$ answers a guess $x^{\prime }\in \left\{0,1\right\}$ and is winning when $x=x^{\prime }$. $A_{3}$’s advantage is defined as $Adv_{IBME-ET,A_{3}}^{ANON-ID-CCA}\left(\lambda \right)=|$Pr$\left[x=x^{\prime }\right]-\frac{1}{2}|.$*

In the above game, the constraint is that $A_{3}$ cannot ask the following queries:

- $O_{SKGen}\left({snd}_{0}^{*}\right)$, $O_{SKGen}\left({snd}_{1}^{*}\right)$, $O_{Enc}\left({snd}_{0}^{*},\rho_{0}^{*},*\right)$ and $O_{Enc}\left({snd}_{1}^{*},\rho_{1}^{*},*\right)$.
- $O_{RKGen}\left(\rho_{0}^{*}\right)$, $O_{RKGen}\left(\rho_{1}^{*}\right)$, $O_{Auth}\left({snd}_{0}^{*},\rho_{0}^{*}\right)$ and $O_{Auth}\left({snd}_{1}^{*},\rho_{1}^{*}\right)$.
- $O_{Dec}\left(\rho_{0}^{*},{snd}_{0}^{*},C^{*}\right)$, $O_{Dec}\left(\rho_{1}^{*},{snd}_{1}^{*},C^{*}\right)$.

**Definition** **4** (sUF-ID-CMA)**.** *Regarding $A_{4}$, the IBME-ET scheme meets sUF-ID-CMA security when no PPT $A_{4}$ is winning the game below with a non-negligible advantage:*

*1*. *Setup: Same as Definition 1.*
*2*. *Queries: $A_{4}$ can issue queries to the oracles: $O_{SKGen}$, $O_{RKGen}$, $O_{Auth}$, $O_{Enc}$, $O_{Dec}$.*
*3*. Forgery: $A_{4}$ answers a triple $\left({snd}^{*},\rho^{*},C^{*}\right)$. $A_{4}$ is winning when $m^{*}=Dec(pp,dk_{\rho^{*}}$, ${snd}^{*},C^{*})\neq ⊥$. $A_{4}$’s advantage is defined as $Adv_{IBME-ET,A_{4}}^{sUF-ID-CMA}\left(\lambda \right)=$Pr$[A_{4}$ wins].

In the above game, the constraint is that $A_{4}$ cannot make the following queries: $O_{SKGen}\left({snd}^{*}\right)$ and $O_{RKGen}\left(\rho^{*}\right)$. Furthermore, $C^{*}$ cannot be an output of $O_{Enc}\left({snd}^{*},\rho^{*},*\right)$.

## 4. Our Construction

The IBME-ET scheme is concretely constructed as below:

- *Setup*(λ): The following steps are taken:
  1. Randomly select the generators g∈G along with $\hat{g}\in \hat{G}$.
  2. Randomly select numbers $s,\alpha ,\beta_{0},\beta_{1}\in Z_{q}^{*}$, and set $g_{1}=g^{\alpha }$, $f=g^{\beta_{0}}$, $\hat{f}={\hat{g}}^{\beta_{0}}$, $h=g^{\beta_{1}}$, $\hat{h}={\hat{g}}^{\beta_{1}}$.
  3. Secure hash functions are defined: $H:G_{T}\to Z_{q}^{*}$, $H_{1}:{\left\{0,1\right\}}^{*}\to G$, $H_{2}:{\left\{0,1\right\}}^{*}\to \hat{G}$, $H_{3}:{\left\{0,1\right\}}^{*}\to \hat{G}$, $H_{4}:G_{T}\to Z_{q}^{*}$, $H_{5}:{\left\{0,1\right\}}^{\lambda +l}\to Z_{q}^{*}$, $H_{6}:G_{T}^{2}\times G^{3}\to {\left\{0,1\right\}}^{\lambda +l}$, $H_{7}:{\left\{0,1\right\}}^{\lambda }\to \hat{G}$, and $H_{8}:G_{T}\to \hat{G}$.
  4. Return the master key mk along with the system parameters pp, where
    mk=(s,α),
    $$pp=(G,g,\hat{g},g_{1},f,h,\hat{f},\hat{h},H,H_{1},H_{2},H_{3},H_{4},H_{5},H_{6},H_{7},H_{8}).$$
- *SKGen*(pp,mk,σ): Let mk=(s,α). This algorithm produces the encryption key $ek_{\sigma }={H_{1}\left(\sigma \right)}^{s}.$
- *RKGen*(pp,mk,ρ): Let mk=(s,α). This algorithm produces the decryption key $dk_{\rho }=\left(d_{1},d_{2},d_{3}\right)=\left({H_{3}\left(\rho \right)}^{s},{H_{2}\left(\rho \right)}^{\alpha },{H_{3}\left(\rho \right)}^{\alpha }\right)$.
- *Enc*($pp,ek_{\sigma },rcv,m$): Let rcv=ρ and $m\in {\left\{0,1\right\}}^{\lambda }$. The ciphertext $C=(C_{0},C_{1},C_{2},C_{3},C_{4})$ is calculated as below:
  1. Randomly select $r\in Z_{q}^{*}$ and $k\in {\left\{0,1\right\}}^{l}$, and calculate $R=H_{5}\left(m,k\right)$.
  2. Calculate $\eta =e(ek_{\sigma },H_{3}\left(\rho )\right)$, $\omega_{1}={e(g_{1},H_{2}\left(\rho )\right)}^{r\cdot H_{4}\left(\eta \right)}$ and $\omega_{2}={e(g_{1},H_{3}\left(\rho )\right)}^{r\cdot H_{4}\left(\eta \right)}$.
  3. Calculate the following numbers:
    $$\begin{array}{c} \left\{\begin{array}{c} C_{0}=g^{R},\\ C_{1}=g^{r},\\ C_{2}={\left(fh^{H(\eta )}\right)}^{r},\\ C_{3}=\left(m\|k\right)⊕H_{6}\left(\omega_{1},\eta ,C_{0},C_{1},C_{2}\right),\\ C_{4}=H_{7}{\left(m\right)}^{R}\cdot H_{8}\left(\omega_{2}\right). \end{array}\right. \end{array}$$
- Dec($pp,dk_{\rho },snd,C$): Let $dk_{\rho }=\left(d_{1},d_{2},d_{3}\right)$, snd=σ. The following steps are taken:
  1. Calculate $\eta =e\left(H_{1}(\sigma \right),d_{1}),\;\omega_{1}=e\left(C_{1},d_{2}^{H_{4}\left(\eta \right)}\right)$ and $\omega_{2}=e\left(C_{1},d_{3}^{H_{4}\left(\eta \right)}\right)$.
  2. Obtain $m^{\prime }\|k^{\prime }$ by computing $C_{3}⊕H_{6}\left(\omega_{1},\eta ,C_{0},C_{1},C_{2}\right)$.
  3. Calculate $R^{\prime }=H_{5}\left(m^{\prime },k^{\prime }\right)$.
  4. If $C_{0}=g^{R^{\prime }}$ and $C_{4}=H_{7}{\left(m^{\prime }\right)}^{R^{\prime }}\cdot H_{8}\left(\omega_{2}\right)$ hold, answer $m^{\prime }$; otherwise, answer ⊥.
- *Auth*($pp,snd,dk_{\rho }$): Let $dk_{\rho }=\left(d_{1},d_{2},d_{3}\right)$ and snd=σ. The following steps are taken:
  1. Randomly select $y\in Z_{q}^{*}$, and calculate $\eta =e\left(H_{1}(\sigma \right),d_{1})$.
  2. Return the trapdoor $td_{\left(snd,\rho \right)}=\left(y_{1},y_{2}\right)=\left(d_{3}^{H_{4}\left(\eta \right)}{\left(\hat{f}{\hat{h}}^{H(\eta )}\right)}^{y},\;{\hat{g}}^{y}\right)$.
- *Test*($pp,C_{\left(\sigma_{A},{rcv}_{A}\right)},td_{\left({snd}_{A},\rho_{A}\right)},C_{\left(\sigma_{B},{rcv}_{B}\right)},td_{\left({snd}_{B},\rho_{B}\right)}$): Let $C_{\left(\sigma_{A},{rcv}_{A}\right)}=(C_{\sigma_{A},{rcv}_{A},0},$$C_{\sigma_{A},{rcv}_{A},1}$, $C_{\sigma_{A},{rcv}_{A},2},$$C_{\sigma_{A},{rcv}_{A},3},C_{\sigma_{A},{rcv}_{A},4})$, $td_{\left({snd}_{A},\rho_{A}\right)}=(y_{{snd}_{A},\rho_{A},1},$$y_{{snd}_{A},\rho_{A},2})$, $C_{\left(\sigma_{B},{rcv}_{B}\right)}=(C_{\sigma_{B},{rcv}_{B},0}$, $C_{\sigma_{B},{rcv}_{B},1},C_{\sigma_{B},{rcv}_{B},2},$$C_{\sigma_{B},{rcv}_{B},3},C_{\sigma_{B},{rcv}_{B},4})$ and $td_{\left({snd}_{B},\rho_{B}\right)})=\left(y_{{snd}_{B},\rho_{B},1},y_{{snd}_{B},\rho_{B},2}\right)$. The following steps are taken:
  1. Calculate
    $$\omega_{A,2}=e(C_{\sigma_{A},{rcv}_{A},1},y_{snd_{A},\rho_{A},1})/e(C_{\sigma_{A},{rcv}_{A},2},y_{snd_{A},\rho_{A},2}),$$
    $$\omega_{B,2}=e(C_{\sigma_{B},{rcv}_{B},1},y_{snd_{B},\rho_{B},1})/e(C_{\sigma_{B},{rcv}_{B},2},y_{snd_{B},\rho_{B},2}).$$
  2. Calculate
    $$K_{A}=C_{\sigma_{A},{rcv}_{A},4}/H_{8}\left(\omega_{A,2}\right),$$
    $$K_{B}=C_{\sigma_{B},{rcv}_{B},4}/H_{8}\left(\omega_{B,2}\right).$$
  3. Check whether $e\left(C_{\sigma_{A},{rcv}_{A},0},K_{B}\right)=e\left(C_{\sigma_{B},{rcv}_{B},0},K_{A}\right)$ holds. When it holds, answer 1 or 0 otherwise.

**Correctness:** The proposed scheme is correct in accordance with the correctness definition:

1. Regarding Condition 1, when σ=snd and ρ=rcv, we have
  $$\begin{array}{cc} & \eta =e(ek_{\sigma },H_{3}\left(\rho )\right)=e\left(H_{1}\left(\sigma \right),H_{3}{\left(\rho \right))}^{s}=e\left(H_{1}(\sigma \right),d_{1}\right),\\ & \omega_{1}={e(g_{1},H_{2}\left(\rho )\right)}^{r\cdot H_{4}\left(\eta \right)}=e{\left(g,H_{2}\left(\rho \right)\right)}^{r\alpha \cdot H_{4}\left(\eta \right)}=e\left(C_{1},d_{2}^{H_{4}\left(\eta \right)}\right),\\ & C_{3}⊕H_{6}\left(\omega_{1},\eta ,C_{0},C_{1},C_{2}\right)=\left(m\|k\right)⊕H_{6}\left(\omega_{1},\eta ,C_{0},C_{1},C_{2}\right)⊕H_{6}\left(\omega_{1},\eta ,C_{0},C_{1},C_{2}\right)=m\|k. \end{array}$$
  Thus, when σ=snd and ρ=rcv, $Dec(pp,dk_{\rho },snd,Enc\left(pp,ek_{\sigma },rcv,m\right))=m$ always holds.
2. Regarding Condition 2, if $\sigma_{A}={snd}_{A}\;\wedge {rcv}_{A}=\rho_{A}$$\wedge \;\sigma_{B}={snd}_{B}\wedge {rcv}_{B}=\rho_{B}$$\wedge \;m_{A}=m_{B}$, we have
  $$\begin{array}{cc} \frac{e(C_{\sigma_{A},\rho_{A},1},y_{\sigma_{A},\rho_{A},1})}{e(C_{\sigma_{A},\rho_{A},2},y_{\sigma_{A},\rho_{A},2})} & =\frac{e(g^{r_{A}},d_{A,3}^{H_{4}\left(\eta_{A}\right)}{\left(\hat{f}{\hat{h}}^{H(\eta_{A})}\right)}^{y_{A}})}{e({\left(fh^{H(\eta_{A})}\right)}^{r_{A}},{\hat{g}}^{y_{A}})}=\frac{e\left(g^{r_{A}},d_{A,3}^{H_{4}\left(\eta_{A}\right)}\right)\cdot e{\left(g,\hat{f}{\hat{h}}^{H(\eta_{A})}\right)}^{r_{A}y_{A}}}{e{\left(fh^{H(\eta_{A})},\hat{g}\right)}^{r_{A}y_{A}}}\\ & =\frac{e\left(g^{r_{A}},d_{A,3}^{H_{4}\left(\eta_{A}\right)}\right)\cdot e{\left(g,{\hat{g}}^{\beta_{0}+\beta_{1}H\left(\eta_{A}\right)}\right)}^{r_{A}y_{A}}}{e{\left(g^{\beta_{0}+\beta_{1}H\left(\eta_{A}\right)},\hat{g}\right)}^{r_{A}y_{A}}}=e\left(g^{r_{A}},d_{A,3}^{H_{4}\left(\eta_{A}\right)}\right)\\ & =e{\left(g,H_{3}\left(\rho_{A}\right)\right)}^{r_{A}\alpha \cdot H_{4}\left(\eta_{A}\right)}={e(g_{1},H_{3}\left(\rho_{A})\right)}^{r_{A}\cdot H_{4}\left(\eta_{A}\right)}=\omega_{A,2},\\ \frac{e(C_{\sigma_{B},\rho_{B},1},y_{\sigma_{B},\rho_{B},1})}{e(C_{\sigma_{B},\rho_{B},2},y_{\sigma_{B},\rho_{B},2})} & =\frac{e(g^{r_{B}},d_{B,3}^{H_{4}\left(\eta_{B}\right)}{\left(\hat{f}{\hat{h}}^{H(\eta_{B})}\right)}^{y_{B}})}{e({\left(fh^{H(\eta_{B})}\right)}^{r_{B}},{\hat{g}}^{y_{B}})}=\frac{e\left(g^{r_{B}},d_{B,3}^{H_{4}\left(\eta_{B}\right)}\right)\cdot e{\left(g,\hat{f}{\hat{h}}^{H(\eta_{B})}\right)}^{r_{B}y_{B}}}{e{\left(fh^{H(\eta_{B})},\hat{g}\right)}^{r_{B}y_{B}}}\\ & =\frac{e\left(g^{r_{B}},d_{B,3}^{H_{4}\left(\eta_{B}\right)}\right)\cdot e{\left(g,{\hat{g}}^{\beta_{0}+\beta_{1}H\left(\eta_{B}\right)}\right)}^{r_{B}y_{B}}}{e{\left(g^{\beta_{0}+\beta_{1}H\left(\eta_{B}\right)},\hat{g}\right)}^{r_{B}y_{B}}}=e\left(g^{r_{B}},d_{B,3}^{H_{4}\left(\eta_{B}\right)}\right)\\ & =e{\left(g,H_{3}\left(\rho_{B}\right)\right)}^{r_{B}\alpha \cdot H_{4}\left(\eta_{B}\right)}={e(g_{1},H_{3}\left(\rho_{B})\right)}^{r_{B}\cdot H_{4}\left(\eta_{B}\right)}=\omega_{B,2}. \end{array}$$
  $$\begin{array}{cc} \; & K_{A}=\frac{C_{\sigma_{A},\rho_{A},4}}{H_{8}\left(\omega_{A,2}\right)}=\frac{H_{7}{\left(m_{A}\right)}^{R_{A}}\cdot H_{8}\left(\omega_{A,2}\right)}{H_{8}\left(\omega_{A,2}\right)}=H_{7}{\left(m_{A}\right)}^{R_{A}},\\ \; & K_{B}=\frac{C_{\sigma_{B},\rho_{B},4}}{H_{8}\left(\omega_{B,2}\right)}=\frac{H_{7}{\left(m_{B}\right)}^{R_{B}}\cdot H_{8}\left(\omega_{B,2}\right)}{H_{8}\left(\omega_{B,2}\right)}=H_{7}{\left(m_{B}\right)}^{R_{B}},\\ \; & e\left(C_{\sigma_{A},\rho_{A},0},K_{B}\right)=e\left(g^{R_{A}},H_{7}{\left(M_{B}\right)}^{R_{B}}\right)=e{\left(g,H_{7}\left(M_{B}\right)\right)}^{R_{A}R_{B}},\\ \; & e\left(C_{\sigma_{B},\rho_{B},0},K_{A}\right)=e\left(g^{R_{B}},H_{7}{\left(M_{A}\right)}^{R_{A}}\right)=e{\left(g,H_{7}\left(M_{A}\right)\right)}^{R_{A}R_{B}}. \end{array}$$
  If $\sigma_{A}={snd}_{A}\;\wedge \;{rcv}_{A}=\rho_{A}$$\wedge \;\sigma_{B}={snd}_{B}\wedge \;{rcv}_{B}=\rho_{B}$$\wedge \;m_{A}=m_{B}$, then $e\left(C_{\sigma_{A},\rho_{A},0},K_{B}\right)=e\left(C_{\sigma_{B},\rho_{B},0},K_{A}\right)$, so $\;Test(pp,C_{\left(\sigma_{A},{rcv}_{A}\right)},td_{\left(snd_{A},\rho_{A}\right)},C_{\left(\sigma_{B},{rcv}_{B}\right)},td_{\left(snd_{B},\rho_{B}\right)})=1;$ otherwise, Pr[Test(pp,$C_{\left(\sigma_{A},{rcv}_{A}\right)},td_{\left(snd_{A},\rho_{A}\right)},C_{\left(\sigma_{B},{rcv}_{B}\right)},td_{\left(snd_{B},\rho_{B}\right)})=1]$ is negligible due to the hash functions $H_{7}$ and $H_{8}$ being collision-resistant.

## 5. Security Analysis

In the random oracle model, we used the method of proof by contradiction to show that if the BDH assumption and Gap-BDH assumption introduced in the preliminaries (see Section 2) hold, and our proposed IBME-ET scheme can meet confidentiality, authenticity, and anonymity in cryptography [30,31,32].

According to our IBME-ET scheme, given the ciphertext *C*, we have the following observations:

- To reveal the message *m*, it is necessary to calculate $\omega_{1}={e(g_{1},H_{2}\left(\rho )\right)}^{r\cdot H_{4}\left(\eta \right)}$.
- To obtain $H_{7}{\left(m\right)}^{R}$, which is used for the equality test, it is necessary to calculate $\omega_{2}={e(g_{1},H_{3}\left(\rho )\right)}^{r\cdot H_{4}\left(\eta \right)}$.
- To distinguish the identities of the sender and the receiver concealed in the ciphertext, it is necessary to calculate $\eta =e(ek_{\sigma },H_{3}\left(\rho )\right)=e(H_{1}\left(\sigma \right),H_{3}{\left(\rho \right))}^{s}$.
- To fake any legitimate ciphertext pertaining to the sender σ and the receiver ρ, it is necessary to calculate $\eta =e(ek_{\sigma },H_{3}\left(\rho )\right)=e(H_{1}\left(\sigma \right),H_{3}{\left(\rho \right))}^{s}$.

Note that, regarding to the confidentiality, anonymity, and authenticity of the IBME-ET, four security models are defined by considering four distinct types of adversaries (see Section 3.3). The security proof of our scheme can be outlined as follows:

As for the confidentiality, we first used the BDH assumption to prove that our proposal meets IND-ID-CCA security regarding the Type-I adversary $A_{1}$. Given a BDH assumption instance $\left(g,g^{a},g^{c},\hat{g},{\hat{g}}^{a},{\hat{g}}^{b}\right)$, we generated a simulated scheme B and interacted with $A_{1}$ by following the IND-ID-CCA security model defined in Section 3.3. B simulates the oracles $O_{SKGen}$, $O_{RKGen}$, $O_{Auth}$, and $O_{Dec}$ to answer $A_{1}$’s queries and preserves the $L_{H}$ and $L_{H_{i}}\left(i=1,2,3,5,6,7,8\right)$ lists to simulate the random oracles $O_{H}$ and $O_{H_{i}}\left(i=1,2,3,5,6,7,8\right)$. In the challenge phase, $A_{1}$ sends identities $\sigma^{*},{rcv}^{*}$ and equal-length messages $m_{0}^{*},m_{1}^{*}$ to B. Let ${rcv}^{*}=\rho^{*}$. B randomly selects x∈{0,1} and answers the challenge ciphertext $C^{*}=\left(C_{0}^{*},C_{1}^{*},C_{2}^{*},C_{3}^{*},C_{4}^{*}\right)=Enc\left(pp,ek_{\sigma^{*}},\rho^{*},m_{x}^{*}\right)$ to $A_{1}$. In the simulation, the challenge ciphertext implicitly sets $\omega_{1}^{*}=e{\left(g,\hat{g}\right)}^{abcv^{*}\cdot H_{4}\left(\eta^{*}\right)}$, $\omega_{2}^{*}=e{\left(g,\hat{g}\right)}^{abct^{*}\cdot H_{4}\left(\eta^{*}\right)}$, $H_{6}(\omega_{1}^{*},\eta^{*},C_{0}^{*},$$C_{1}^{*},C_{2}^{*})=\left(m_{x}\|k\right)⊕C_{3}^{*}$, $H_{8}\left(\omega_{2}^{*}\right)$$=\frac{C_{4}^{*}}{H_{7}{\left(m_{x}\right)}^{R}}$, where $g_{1}=g^{a}$, $H_{2}\left(\rho^{*}\right)={\hat{g}}^{bv^{*}}$, $H_{3}\left(\rho^{*}\right)={\hat{g}}^{bt^{*}}$, $H_{1}\left(\sigma^{*}\right)=g^{u^{*}}$, $ek_{\sigma^{*}}=g^{su^{*}}$, $\eta^{*}=e{\left(g,\hat{g}\right)}^{bsu^{*}t^{*}}$, $C_{0}^{*}=g^{R}$, $C_{1}^{*}=g^{c}$, and $C_{2}^{*}=g^{\beta_{0}^{\prime }c}$. Finally, in the guess phase, $A_{1}$ outputs a guess $x^{\prime }\in \left\{0,1\right\}$. The advantage of $A_{1}$ for breaking our proposal is defined as ϵ=|Pr$\left[x=x^{\prime }\right]-\frac{1}{2}|.$ If ϵ is non-negligible, then the tuple $\left[\omega_{1}^{*},\eta^{*},C_{0}^{*},C_{1}^{*},C_{2}^{*},\delta^{*}\right]$ is documented in $L_{H_{6}}$ with non-negligible probability. If B selects the right tuple from $L_{H_{6}}$, B can return the BDH instance solution ${\omega_{1}^{*}}^{{\left(v^{*}H_{4}\left(\eta^{*}\right)\right)}^{-1}}$$\left(=e{\left(g,\hat{g}\right)}^{abc}\right)$. As a result, the BDH assumption can be addressed by B with non-negligible advantage if $A_{1}$ is able to break our proposal with non-negligible advantage.

Subsequently, as for the confidentiality, we used the BDH assumption to prove that our proposal meets OW-ID-CCA security regarding the Type-II adversary $A_{2}$. Given a BDH assumption instance $(g,g^{a},g^{c},$ $\hat{g},{\hat{g}}^{a},{\hat{g}}^{b})$, we generated a simulated scheme B and interacted with $A_{2}$ by following the OW-ID-CCA security model defined in Section 3.3. B simulates the oracles $O_{SKGen}$, $O_{RKGen}$, $O_{Auth}$, and $O_{Dec}$ to answer $A_{2}$’s queries and preserves the $L_{H}$ and $L_{H_{i}}\left(i=1,2,3,5,6,7,8\right)$ lists to simulate the random oracles $O_{H}$ and $O_{H_{i}}\left(i=1,2,3,5,6,7,8\right)$. In the challenge phase, $A_{2}$ sends identities $\sigma^{*},{rcv}^{*}$ to B. Let ${rcv}^{*}=\rho^{*}$. B randomly chooses a message $m^{*}\in {\left\{0,1\right\}}^{\lambda }$ and answers the challenge ciphertext $C^{*}=\left(C_{0}^{*},C_{1}^{*},C_{2}^{*},C_{3}^{*},C_{4}^{*}\right)=Enc\left(pp,ek_{\sigma^{*}},\rho^{*},m^{*}\right)$ to $A_{2}$. In the simulation, the challenge ciphertext implicitly sets $\omega_{1}^{*}=e{\left(g,\hat{g}\right)}^{abcv^{*}\cdot H_{4}\left(\eta^{*}\right)}$, $H_{6}\left(\omega_{1}^{*},\eta^{*},C_{0}^{*},C_{1}^{*},C_{2}^{*}\right)$$=\left(m^{*}\|k\right)⊕C_{3}^{*}$, where $g_{1}=g^{a}$, $H_{2}\left(\rho^{*}\right)={\hat{g}}^{bv^{*}}$, $H_{3}\left(\rho^{*}\right)={\hat{g}}^{t^{*}}$, $H_{1}\left(\sigma^{*}\right)=g^{u^{*}}$, $ek_{\sigma^{*}}=g^{su^{*}}$, $\eta^{*}=e{\left(g,\hat{g}\right)}^{bsu^{*}t^{*}}$, $C_{0}^{*}=g^{R}$, $C_{1}^{*}=g^{c}$, $C_{2}^{*}=g^{\beta_{0}^{\prime }c}$, and $C_{4}^{*}={H_{7}\left(m^{*}\right)}^{R}\cdot H_{8}\left(e(g^{c},{\hat{g}}^{at^{*}\cdot H_{4}\left(\eta^{*}\right)})\right)$. Finally, in the guess phase, $A_{2}$ outputs a guess $m^{\prime }$. The advantage of $A_{2}$ for breaking our proposal is defined as ϵ=|Pr$\left[m^{*}=m^{\prime }]\right|$. If ϵ is non-negligible, then the tuple $\left[\omega_{1}^{*},\eta^{*},C_{0}^{*},C_{1}^{*},C_{2}^{*},\delta^{*}\right]$ is documented in $L_{H_{6}}$ with non-negligible probability. If B selects the right tuple from $L_{H_{6}}$, B can return the BDH instance solution ${\omega_{1}^{*}}^{{\left(v^{*}H_{4}\left(\eta^{*}\right)\right)}^{-1}}\left(=e{\left(g,\hat{g}\right)}^{abc}\right)$. As a result, the BDH assumption can be addressed by B with non-negligible advantage if $A_{2}$ is able to break our proposal with non-negligible advantage.

As for the anonymity, we used the Gap-BDH assumption to prove that our proposal meets ANON-ID-CCA security regarding the Type-III adversary $A_{3}$. Given a Gap-BDH assumption instance $(g,g^{a},g^{c},\hat{g},{\hat{g}}^{a},{\hat{g}}^{b},$ $O_{\mathrm{DBDH}})$, we generated a simulated scheme B and interacted with $A_{3}$ by following the ANON-ID-CCA security model defined in Section 3.3. B simulates the oracles $O_{H}$, $O_{H_{i}}\left(i=1,2,3,4,5,6,7,8\right)$, $O_{SKGen}$, $O_{RKGen}$, $O_{Auth}$, $O_{Enc}$, and $O_{Dec}$ to answer $A_{3}$’s queries. In the challenge phase, $A_{3}$ sends identities $\left({snd}_{0}^{*},\rho_{0}^{*}\right)$, $\left({snd}_{1}^{*},\rho_{1}^{*}\right)$ and a message $m^{*}$ to B. Let ${snd}_{0}^{*}=\sigma_{0}^{*}$, ${snd}_{1}^{*}=\sigma_{1}^{*}$. B randomly chooses x∈{0,1} and answers the challenge ciphertext $C^{*}=\left(C_{0}^{*},C_{1}^{*},C_{2}^{*},C_{3}^{*},C_{4}^{*}\right)=Enc\left(pp,ek_{\sigma_{x}^{*}},\rho_{x}^{*},m^{*}\right)$ and the challenge trapdoor $td_{\left(\sigma_{x}^{*},\rho_{x}^{*}\right)}=\left(y_{1},y_{2}\right)=Auth\left(pp,\sigma_{x}^{*},dk_{\rho_{x}^{*}}\right)$ to $A_{3}$. In the simulation, the challenge ciphertext implicitly sets $\eta^{*}=e{\left(g,\hat{g}\right)}^{abcu_{x}^{*}t_{x}^{*}}$, $\omega_{1}^{*}=e{\left(g^{a\alpha^{\prime }},{\hat{g}}^{b}\right)}^{r\Omega v_{x}^{*}}$, $C_{3}^{*}=\left(m^{*}\|k\right)⊕H_{6}\left(\omega_{1}^{*},\eta^{*},C_{0}^{*},C_{1}^{*},C_{2}^{*}\right)$, where $g_{1}=g^{a\alpha^{\prime }}$, $H_{1}\left(\sigma_{0}^{*}\right)=g^{cu_{i_{x}^{*}}}$, $H_{2}\left(\rho_{x}^{*}\right)={\hat{g}}^{bv_{j_{t}^{*}}}$, $H_{3}\left(\rho_{x}^{*}\right)={\hat{g}}^{bv_{t_{x}^{*}}}$, $\omega_{2}^{*}=e{\left(g^{a\alpha^{\prime }},{\hat{g}}^{b}\right)}^{r{\tilde{\Omega }}_{xx}}$, $H\left(\eta^{*}\right)=I=I_{xx}$, $H_{4}\left(\eta^{*}\right)=\Omega =\frac{{\tilde{\Omega }}_{xx}}{t_{x}^{*}}$, $C_{0}^{*}=g^{R}$, $C_{1}^{*}=g^{r}$, $C_{2}^{*}={\left(fh^{I}\right)}^{r}$, and $C_{4}^{*}=H_{7}{\left(m^{*}\right)}^{R}\cdot H_{8}\left(\omega_{2}^{*}\right)$ Furthermore, the challenge trapdoor implicitly sets $y=\tilde{y}-bz$, where $z=\frac{t_{x}\alpha^{\prime }\Omega }{\beta_{1}I}=\frac{\alpha^{\prime }{\tilde{\Omega }}_{xx}}{\beta_{1}I}$, $y_{1}={\hat{g}}^{\beta_{0}\left(\tilde{y}-bz\right)}{\hat{g}}^{a\beta_{1}I\tilde{y}}$, $y_{2}={\hat{g}}^{\tilde{y}-bz}$. Finally, in the guess phase, $A_{3}$ outputs a guess $x^{\prime }\in \left\{0,1\right\}$. The advantage of $A_{3}$ for breaking our proposal is defined as ϵ=|Pr$\left[x=x^{\prime }\right]-\frac{1}{2}|.$ If ϵ is non-negligible, $\eta^{*}=e{\left(g,\hat{g}\right)}^{abcu_{x}^{*}t_{x}^{*}}$ has been queried to $O_{H}$ with non-negligible probability. With $O_{\mathrm{DBDH}}(g,g^{a},g^{c},\hat{g},$ ${\hat{g}}^{a},{\hat{g}}^{b},{\eta^{*}}^{{\left(u_{i_{x}^{*}}t_{j_{x}^{*}}\right)}^{-1}})=1$, B can return the Gap-BDH instance solution ${\eta^{*}}^{{\left(u_{i_{x}^{*}}t_{j_{x}^{*}}\right)}^{-1}}\;\left(=e{\left(g,\hat{g}\right)}^{abc}\right)$. As a result, the Gap-BDH assumption can be addressed by B with non-negligible advantage if $A_{3}$ is able to break our proposal with non-negligible advantage.

As for the authenticity, we used the Gap-BDH assumption to prove that our proposal meets sUF-ID-CMA security regarding the Type-IV adversary $A_{4}$. Given a Gap-BDH assumption instance $(g,g^{a},g^{c},\hat{g},{\hat{g}}^{a},{\hat{g}}^{b},$ $O_{\mathrm{DBDH}})$, we generated a simulated scheme B and interacted with $A_{4}$ by following the sUF-ID-CMA security model defined in Section 3.3. B simulates the oracles $O_{H}$, $O_{H_{i}}\left(i=1,2,3,4,5,6,7,8\right)$, $O_{SKGen}$, $O_{RKGen}$, $O_{Auth}$, $O_{Enc}$, and $O_{Dec}$ to answer $A_{4}$’s queries. In the simulation, the following numbers are implicitly set $\eta^{*}=e{\left(g,\hat{g}\right)}^{abc}$, where $H_{1}\left(\sigma^{*}\right)=g^{c}$, $H_{3}\left(\rho^{*}\right)={\hat{g}}^{b}$, $H\left(\eta^{*}\right)=I^{*}$, $H_{4}\left(\eta^{*}\right)=\Omega^{*}$. In the forgery phase, $A_{4}$ outputs a triple $\left(snd^{*},\rho^{*},C^{*}\right)$, where $snd^{*}=\sigma^{*}$ and $C^{*}=\left(C_{0}^{*},C_{1}^{*},C_{2}^{*},C_{3}^{*},C_{4}^{*}\right)$. If $m^{*}=Dec\left(pp,dk_{\rho^{*}},\sigma^{*},C^{*}\right)\neq ⊥$, $A_{4}$ wins. The advantage of $A_{4}$ for breaking our proposal is defined as ϵ=Pr$\left[A_{4}\mathrm{wins}\right]$. With ϵ and the lemma on the relationship between the chosen-identity attack and given identity attack [33], if ϵ is non-negligible, $\eta^{*}=e{\left(g,\hat{g}\right)}^{abc}$ has been queried to $O_{H}$ with non-negligible probability. Then, $O_{\mathrm{DBDH}}(g,g^{a},g^{c},\hat{g},$${\hat{g}}^{a},{\hat{g}}^{b},\eta^{*})=1$, B can return the Gap-BDH instance solution $\eta^{*}\;\left(=e{\left(g,\hat{g}\right)}^{abc}\right)$. As a result, the Gap-BDH assumption can be addressed by B with non-negligible advantage if $A_{4}$ is able to break our proposal with non-negligible advantage.

**Theorem** **1.** *For any $A_{1}$, our IBME-ET scheme meets IND-ID-CCA security on the basis of the BDH assumption.*

*More precisely, if $A_{1}$ is able to break our proposal with the advantage ϵ, we can conceive of a PPT algorithm B to address the BDH assumption with the advantage $\epsilon^{\prime }\geq \frac{1}{q_{H_{6}}}\left(\frac{\epsilon }{q_{H_{1}}q_{H_{2}}}-\frac{q_{D}}{2^{\lambda +l}}-\frac{q_{H_{8}}}{q}\right),$ where $q_{H_{i}}\left(i=1,2,6,8\right)$ and $q_{D}$ denote the numbers of different queries to $O_{H_{i}}\left(i=1,2,6,8\right)$ and $O_{Dec}$, respectively.*

**Proof.**  Given a BDH assumption instance $\left(g,g^{a},g^{c},\hat{g},{\hat{g}}^{a},{\hat{g}}^{b}\right)$, the task of B is to calculate $e{\left(g,\hat{g}\right)}^{abc}$ by interacting with $A_{1}$ as below:

(1) *Setup*: B randomly selects $i^{*}\in \left\{1,2,\cdots ,q_{H_{1}}\right\}$, $j^{*}\in \left\{1,2,\cdots ,q_{H_{2}}\right\}$. B randomly chooses $I^{*},s,\beta_{0}^{\prime },\beta_{1}^{\prime }\in Z_{q}^{*}$, calculates $g_{1}=g^{a}$, $f=g^{\beta_{0}^{\prime }-a\beta_{1}^{\prime }I^{*}}$, $h=g^{a\beta_{1}^{\prime }}$, $\hat{f}={\hat{g}}^{\beta_{0}^{\prime }-a\beta_{1}^{\prime }I^{*}}$, and $\hat{h}={\hat{g}}^{a\beta_{1}^{\prime }}$, sets $pp=(G,g,\hat{g},g_{1},f,h,\hat{f},\hat{h},$$H,H_{i}\left(i=1,2,3,4,5,6,7,8\right))$, and delivers this to $A_{1}$ with pp. B implicitly sets mk=(s,a), because B has no knowledge about *a*. B preserves the $L_{H}$ and $L_{H_{i}}\left(i=1,2,3,5,6,7,8\right)$ lists to simulate $O_{H}$ and $O_{H_{i}}\left(i=1,2,3,5,6,7,8\right)$. Afterwards, B randomly selects $u^{*},v^{*},t^{*}\in Z_{q}^{*}$.
(2) *Phase1*: B answers $A_{1}$’s queries.
  - $O_{H}\left(\eta \right)$: When $\eta \neq e{\left(g,\hat{g}\right)}^{bsu^{*}t^{*}}$, B randomly selects $I\in Z_{q}^{*}$, inserts a tuple [η,I] into $L_{H}$, and answers *I*. Otherwise, B answers $I^{*}$.
  - $O_{H_{1}}\left(\sigma_{i}\right)$: Suppose $\sigma_{i}$ as the *i*-th different query. When $i\neq i^{*}$, B randomly selects $u_{i}\in Z_{q}^{*}$, inserts a tuple $\left[\sigma_{i},u_{i}\right]$ into $L_{H_{1}}$, and returns $g^{u_{i}}$. Otherwise, B has $u_{i^{*}}=u^{*}$, inserts a tuple $\left[\sigma_{i^{*}},u_{i^{*}}\right]$ into $L_{H_{1}}$, and returns $g^{u_{i^{*}}}$.
  - $O_{H_{2}}\left(\rho_{j}\right)$: Suppose $\rho_{j}$ as the *j*-th different query. When $j\neq j^{*}$, B randomly selects $v_{j}\in Z_{q}^{*}$, inserts a tuple $\left[\rho_{j},v_{j}\right]$ into $L_{H_{2}}$, and returns ${\hat{g}}^{v_{j}}$. Otherwise, B has $v_{j^{*}}=v^{*}$, inserts a tuple $\left[\rho_{j^{*}},v_{j^{*}}\right]$ into $L_{H_{2}}$, and returns ${\hat{g}}^{bv_{j^{*}}}$.
  - $O_{H_{3}}\left(\rho_{j}\right)$: B performs a simulation algorithm to query $O_{H_{2}}\left(\rho_{j}\right)$. Subsequently, B searches the tuple $\left[\rho_{j},v_{j}\right]$ in $L_{H_{2}}$. When $j\neq j^{*}$, B selects $t_{j}\in Z_{q}^{*}$ randomly, inserts a tuple $\left[\rho_{j},t_{j}\right]$ into $L_{H_{3}}$, and returns ${\hat{g}}^{t_{j}}$. Otherwise, B has $t_{j^{*}}=t^{*}$, inserts a tuple $\left[\rho_{j^{*}},t_{j^{*}}\right]$ into $L_{H_{3}}$, and returns ${\hat{g}}^{bt_{j^{*}}}$.
  - $O_{H_{5}}\left(m,k\right)$: B randomly chooses $R\in Z_{q}^{*}$, inserts a tuple [m,k,R] into $L_{H_{5}}$, and answers *R*.
  - $O_{H_{6}}\left(\omega_{1},\eta ,C_{0},C_{1},C_{2}\right)$: B randomly chooses $\delta \in {\left\{0,1\right\}}^{\lambda +l}$, inserts a tuple $\left[\omega_{1},\eta ,C_{0},C_{1},C_{2},\delta \right]$ into $L_{H_{6}}$, and answers δ.
  - $O_{H_{7}}\left(m\right)$: B randomly selects $h_{7}\in \hat{G}$, inserts a tuple $\left[m,h_{7}\right]$ into $L_{H_{7}}$, and returns $h_{7}$.
  - $O_{H_{8}}\left(\omega_{2}\right)$: B randomly selects $\pi \in \hat{G}$, inserts a tuple $\left[\omega_{2},\pi \right]$ into $L_{H_{8}}$, and returns π.
  - $O_{SKGen}\left(\sigma_{i}\right)$: B performs a simulation algorithm to query $O_{H_{1}}\left(\sigma_{i}\right)$. There is a tuple $\left[\sigma_{i},u_{i}\right]$ in $L_{H_{1}}$. Next, B returns $ek_{\sigma_{i}}=g^{su_{i}}$.
  - $O_{RKGen}\left(\rho_{j}\right)$: B performs a simulation algorithm to query $O_{H_{3}}\left(\rho_{j}\right)$. There are a tuple $\left[\rho_{j},v_{j}\right]$ in $L_{H_{2}}$ and a tuple $\left[\rho_{j},t_{j}\right]$ in $L_{H_{3}}$. When $j\neq j^{*}$, B returns $dk_{\rho_{j}}=\left(d_{1},d_{2},d_{3}\right)=\left({\hat{g}}^{st_{j}},\;{\hat{g}}^{av_{j}},\;{\hat{g}}^{at_{j}}\right)$. Otherwise, B is aborted by failure.
  - $O_{Dec}\left(\rho_{j},snd,C\right)$: Let $snd=\sigma_{i}$. B performs a simulation algorithm to query $O_{H_{3}}\left(\rho_{j}\right)$ and $O_{H_{1}}\left(\sigma_{i}\right)$.
    - When $j\neq j^{*}$, B can query $O_{RKGen}\left(\rho_{j}\right)$ to obtain $dk_{\rho_{j}}$ and returns the outcome of the algorithm $Dec(pp,dk_{\rho_{j}},\sigma_{i},C)$.
    - Otherwise, B can query $O_{SKGen}\left(\sigma_{i}\right)$ to obtain $ek_{\sigma_{i}}$ and calculates $\eta =e(ek_{\sigma_{i}},$$H_{3}\left(\rho_{j}\right))$. For each tuple $\left[\omega_{1},\eta ,C_{0},C_{1},C_{2},\delta \right]$ in $L_{H_{6}}$, B calculates $m^{\prime }\|k^{\prime }=C_{3}⊕\delta$ and calculates $R^{\prime }=H_{5}\left(m^{\prime },k^{\prime }\right)$. If $C_{0}=g^{R^{\prime }}$ and there exists a tuple $\left[\omega_{2},\pi \right]$ in $L_{H_{8}}$ such that $C_{4}=H_{7}{\left(m^{\prime }\right)}^{R^{\prime }}\cdot \pi$ holds, it outputs $m^{\prime }$. Once $L_{H_{8}}$ has no such tuple, B outputs ⊥.
  - $O_{Auth}\left(snd,\rho_{j}\right)$: Let $snd=\sigma_{i}$. B performs a simulation algorithm to query $O_{H_{3}}\left(\rho_{j}\right)$ and $O_{H_{1}}\left(\sigma_{i}\right)$. When $j\neq j^{*}$, B can query $O_{RKGen}\left(\rho_{j}\right)$ to obtain $dk_{\rho_{j}}$, returns $td_{\left(\sigma_{i},\rho_{j}\right)}=Auth\left(pp,\sigma_{i},dk_{\rho_{j}}\right)$. Otherwise, B executes the following operations:
    - When $\left(i,j\right)=\left(i^{*},j^{*}\right)$, B is aborted by failure.
    - Otherwise, $L_{H_{2}}$ has a tuple $\left[\rho_{j^{*}},v_{j^{*}}\right]$ and $L_{H_{3}}$ has a tuple $\left[\rho_{j^{*}},t_{j^{*}}\right]$, and B can query $O_{SKGen}\left(\sigma_{i}\right)$ to obtain $ek_{\sigma_{i}}$, calculates $\eta =e(ek_{\sigma_{i}},H_{3}\left(\rho_{j}\right))$, I=H(η) and $\Omega =H_{4}\left(\eta \right)$, randomly selects $\tilde{y}\in Z_{q}^{*}$, calculates $z=\frac{t_{j^{*}}\Omega }{\beta_{1}^{\prime }\left(I-I^{*}\right)}$, implicitly sets $y=\tilde{y}-bz$, and returns $td_{\left(\sigma_{i},\rho_{j^{*}}\right)}=$$\left(y_{1},y_{2}\right)=({\hat{g}}^{\beta_{0}^{\prime }\left(\tilde{y}-bz\right)}{\hat{g}}^{a\beta_{1}^{\prime }\left(I-I^{*}\right)\tilde{y}},$${\hat{g}}^{\tilde{y}-bz})$. $td_{\left(\sigma_{i},\rho_{j^{*}}\right)}=\left(y_{1},y_{2}\right)$ is a valid random trapdoor according to $\rho_{j^{*}}$ and $\sigma_{i}$, where
      $$\begin{array}{cc} y_{1} & ={\hat{g}}^{\beta_{0}^{\prime }\left(\tilde{y}-bz\right)}{\hat{g}}^{a\beta_{1}^{\prime }\left(I-I^{*}\right)\tilde{y}}={\hat{g}}^{abt_{j^{*}}\cdot \Omega }{\hat{g}}^{\beta_{0}^{\prime }y}{\hat{g}}^{a\beta_{1}^{\prime }\left(I-I^{*}\right)y}=d_{3}^{\Omega }{\hat{g}}^{(\beta_{0}^{\prime }-a\beta_{1}I^{*}+a\beta_{1}^{\prime }I)y}=d_{3}^{\Omega }{\left(\hat{f}{\hat{h}}^{I}\right)}^{y},\\ \;y_{2} & ={\hat{g}}^{\tilde{y}-bz}={\hat{g}}^{y}. \end{array}$$
(3) *Challenge*: $A_{1}$ offers equal-length messages $m_{0}^{*},m_{1}^{*}\in {\left\{0,1\right\}}^{\lambda }$ along with the pair of sender/receiver identities ($\sigma^{*},{rcv}^{*}$) to B. Let ${rcv}^{*}=\rho^{*}$. Afterwards, B utilizes a simulation algorithm to query $O_{H_{1}}\left(\sigma^{*}\right)$ and $O_{H_{3}}\left(\rho^{*}\right)$.
  - When the $i^{*}$-th tuple in $L_{H_{1}}$ is $\left[\sigma^{*},u^{*}\right]$ and the $j^{*}$-th tuple in $L_{H_{2}}$ is $\left[\rho^{*},v^{*}\right]$, B randomly selects x∈{0,1}, $C_{3}^{*}\in {\left\{0,1\right\}}^{\lambda +l}$, $C_{4}^{*}\in \hat{G}$ and $k\in {\left\{0,1\right\}}^{l}$, calculates $ek_{\sigma^{*}}=g^{su^{*}}$, $\eta^{*}=e{\left(g,\hat{g}\right)}^{bsu^{*}t^{*}}$, $R=H_{5}\left(m_{x},k\right)$, $C_{0}^{*}=g^{R}$, $C_{1}^{*}=g^{c}$, and $C_{2}^{*}=g^{\beta_{0}^{\prime }c}$, and then, sends the challenge ciphertext $C^{*}=\left(C_{0}^{*},C_{1}^{*},C_{2}^{*},C_{3}^{*},C_{4}^{*}\right)$ to $A_{1}$.
    The above construction implicitly sets $\omega_{1}^{*}=e{\left(g,\hat{g}\right)}^{abcv^{*}\cdot H_{4}\left(\eta^{*}\right)}$, $\omega_{2}^{*}=$$e{\left(g,\hat{g}\right)}^{abct^{*}\cdot H_{4}\left(\eta^{*}\right)}$, $H_{6}(\omega_{1}^{*},\eta^{*},C_{0}^{*},$$C_{1}^{*},C_{2}^{*})=\left(m_{x}\|k\right)⊕C_{3}^{*}$, $H_{8}\left(\omega_{2}^{*}\right)$$=\frac{C_{4}^{*}}{H_{7}{\left(m_{x}\right)}^{R}}$, where $g^{u^{*}}=H_{1}\left(\sigma^{*}\right)$, ${\hat{g}}^{bv^{*}}=H_{2}\left(\rho^{*}\right)$, ${\hat{g}}^{bt^{*}}=H_{3}\left(\rho^{*}\right)$.
  - Otherwise, B is aborted by failure.
(4) *Phase2*: $A_{1}$ makes queries like in Phase 1.
(5) *Guess*: $A_{1}$ answers a guess $x^{\prime }\in \left\{0,1\right\}$. B randomly selects a tuple $\left[\omega_{1}^{*},\eta^{*},C_{0}^{*},C_{1}^{*},C_{2}^{*},\delta^{*}\right]$ from $L_{H_{6}}$ and returns the BDH instance solution ${\omega_{1}^{*}}^{{\left(v^{*}H_{4}\left(\eta^{*}\right)\right)}^{-1}}\left(=e{\left(g,\hat{g}\right)}^{abc}\right)$.

□

*Analysis*: It is obvious that the simulations of $O_{H}$, $O_{H_{1}}$, $O_{H_{2}}$, $O_{H_{3}}$, $O_{H_{5}}$, and $O_{H_{7}}$ are perfect. Denote the query $O_{H_{6}}\left(e{\left(g,\hat{g}\right)}^{abcv^{*}\cdot H_{4}\left(\eta^{*}\right)},\eta^{*},C_{0}^{*},C_{1}^{*},C_{2}^{*}\right)$ as the event $AskH_{6}^{*}$. Denote the query $O_{H_{8}}\left(e{\left(g,\hat{g}\right)}^{abct^{*}\cdot H_{4}\left(\eta^{*}\right)}\right)$ as the event $AskH_{8}^{*}$. Denote the failure of B to decrypt the legitimate ciphertext in $O_{Dec}$ as the event Derr. Thus, $\mathrm{Pr}\left[Derr\right]\leq \frac{q_{D}}{2^{\lambda +l}}$. Let ${rcv}^{*}=\rho^{*}$. Suppose AbortRK as the event in which B terminates upon the query $O_{RKGen}\left(\rho^{*}\right)$ being issued, AbortAuth as the event in which B terminates upon the query $O_{Auth}\left(\sigma^{*},\rho^{*}\right)$ being issued, and AbortCh as the event in which B terminates in the challenge phase. Clearly, ¬AbortCh implies ¬AbortRK and ¬AbortAuth, because the queries $O_{RKGen}\left(\rho^{*}\right)$ and $O_{Auth}\left(\sigma^{*},\rho^{*}\right)$ cannot be issued. We obtain $\mathrm{Pr}\left[\neg AbortCh\right]\geq \frac{1}{q_{H_{1}}q_{H_{2}}}$.

Define $E=(AskH_{6}^{*}\vee AskH_{8}^{*}\vee Derr)|\neg AbortCh$. There is no greater over $\frac{1}{2}$ advantage that $A_{1}$ will gain in guessing *x* when E does not happen because $O_{H_{6}}$ and $O_{H_{8}}$ are random oracles. $\mathrm{Pr}\left[x=x^{\prime }|\neg E\right]=\frac{1}{2}$. Hence,

$$\begin{array}{cc} \mathrm{Pr}[x=x^{\prime }] & =\mathrm{Pr}\left[x=x^{\prime }|\neg E\right]\mathrm{Pr}\left[\neg E\right]+\mathrm{Pr}\left[x=x^{\prime }|E\right]\mathrm{Pr}\left[E\right]\leq \frac{1}{2}\mathrm{Pr}\left[\neg E\right]+\mathrm{Pr}\left[E\right]=\frac{1}{2}+\frac{1}{2}\mathrm{Pr}\left[E\right]. \end{array}$$

With ϵ, we obtain

$$\begin{array}{cc} \epsilon & =|\mathrm{Pr}\left[x=x^{\prime }\right]-\frac{1}{2}|\leq \mathrm{Pr}\left[E\right]\leq \frac{\mathrm{Pr}\left[AskH_{6}^{*}\right]+\mathrm{Pr}\left[AskH_{8}^{*}\right]+\mathrm{Pr}\left[Derr\right]}{\mathrm{Pr}[\neg AbortCh]}. \end{array}$$

Subsequently, we obtain

$$\begin{array}{cc} \mathrm{Pr}[AskH_{6}^{*}] & \geq \epsilon \mathrm{Pr}\left[\neg AbortCh\right]-\mathrm{Pr}\left[Derr\right]-\mathrm{Pr}\left[AskH_{8}^{*}\right]\geq \frac{\epsilon }{q_{H_{1}}q_{H_{2}}}-\frac{q_{D}}{2^{\lambda +l}}-\frac{q_{H_{8}}}{q}. \end{array}$$

When $AskH_{6}^{*}$ happens, $A_{1}$ can distinguish the simulation of the challenge ciphertext $C^{*}$. Because $O_{H_{6}}\left(e{\left(g,\hat{g}\right)}^{abcv^{*}\cdot H_{4}\left(\eta^{*}\right)},\eta^{*},C_{0}^{*},C_{1}^{*},C_{2}^{*}\right)$ has been documented in $L_{H_{6}}$ with non-negligible probability, B is winning when the right element is selected from $L_{H_{6}}$. Thus, the BDH assumption can be addressed by B with advantage $\epsilon^{\prime }\geq \frac{1}{q_{H_{6}}}\mathrm{Pr}\left[AskH_{6}^{*}\right]\geq \frac{1}{q_{H_{6}}}\left(\frac{\epsilon }{q_{H_{1}}q_{H_{2}}}-\frac{q_{D}}{2^{\lambda +l}}-\frac{q_{H_{8}}}{q}\right).$

**Theorem** **2.** *For any $A_{2}$, our IBME-ET scheme meets OW-ID-CCA security on the basis of the BDH assumption.*

*More precisely, if $A_{2}$ is able to break our proposal with the advantage ϵ, we are able to conceive of a PPT algorithm B to address the BDH assumption with the advantage $\epsilon^{\prime }\geq \frac{1}{q_{H_{6}}}\left(\frac{\epsilon -\frac{1}{2^{\lambda }}}{q_{H_{1}}q_{H_{2}}}-\frac{q_{D}}{2^{\lambda +l}}\right),$ where $q_{H_{i}}\left(i=1,2,6\right)$ and $q_{D}$ denote the numbers of different queries to $O_{H_{i}}\left(i=1,2,6\right)$ and $O_{Dec}$, respectively.*

**Proof.**  Given a BDH assumption instance $\left(g,g^{a},g^{c},\hat{g},{\hat{g}}^{a},{\hat{g}}^{b}\right)$, the task of B is to calculate $e{\left(g,\hat{g}\right)}^{abc}$ by interacting with $A_{2}$ as below:

(1) *Setup*: B executes like in the proof of Theorem 1.
(2) *Phase1*: B answers $A_{2}$’s queries.
  - For $O_{H}\left(\eta \right)$, $O_{H_{1}}\left(\sigma_{i}\right)$, $O_{H_{2}}\left(\rho_{j}\right)$, $O_{H_{5}}\left(m,k\right)$, $O_{H_{6}}\left(\omega_{1},\eta ,C_{0},C_{1},C_{2}\right)$, $O_{H_{7}}\left(m\right)$, and $O_{H_{8}}\left(\omega_{2}\right)$, B executes like in the proof of Theorem 1.
  - $O_{H_{3}}\left(\rho_{j}\right)$: B performs a simulation algorithm to query $O_{H_{2}}\left(\rho_{j}\right)$. Subsequently, B searches the tuple $\left[\rho_{j},v_{j}\right]$ in $L_{H_{2}}$. When $j\neq j^{*}$, B randomly selects $t_{j}\in Z_{q}^{*}$, inserts a tuple $\left[\rho_{j},t_{j}\right]$ into $L_{H_{3}}$, and returns ${\hat{g}}^{t_{j}}$. Otherwise, B sets $t_{j^{*}}=t^{*}$, inserts a tuple $\left[\rho_{j^{*}},t_{j^{*}}\right]$ into $L_{H_{3}}$, and returns ${\hat{g}}^{t_{j^{*}}}$.
  - $O_{SKGen}\left(\sigma_{i}\right)$: B performs a simulation algorithm to query $O_{H_{1}}\left(\sigma_{i}\right)$. There is a tuple $\left[\sigma_{i},u_{i}\right]$ in $L_{H_{1}}$. Next, B returns $ek_{\sigma_{i}}=g^{su_{i}}$.
  - $O_{RKGen}\left(\rho_{j}\right)$: B performs a simulation algorithm to query $O_{H_{3}}\left(\rho_{j}\right)$. There are a tuple $\left[\rho_{j},v_{j}\right]$ in $L_{H_{2}}$ and a tuple $\left[\rho_{j},t_{j}\right]$ in $L_{H_{3}}$. When $j\neq j^{*}$, B returns $dk_{\rho_{j}}=\left(d_{1},d_{2},d_{3}\right)=\left({\hat{g}}^{st_{j}},\;{\hat{g}}^{av_{j}},\;{\hat{g}}^{at_{j}}\right)$. Otherwise, B is aborted by failure.
  - $O_{Dec}\left(\rho_{j},snd,C\right)$: Let $snd=\sigma_{i}$. B performs a simulation algorithm to query $O_{H_{3}}\left(\rho_{j}\right)$ and $O_{H_{1}}\left(\sigma_{i}\right)$.
    - When $j\neq j^{*}$, B can query $O_{RKGen}\left(\rho_{j}\right)$ to obtain $dk_{\rho_{j}}$ and returns the outcome of the algorithm $Dec(pp,dk_{\rho_{j}},\sigma_{i},C)$.
    - Otherwise, B can query $O_{SKGen}\left(\sigma_{i}\right)$ to obtain $ek_{\sigma_{i}}$ and calculates $\eta =e(ek_{\sigma_{i}},H_{3}\left(\rho_{j}\right))$. For each tuple $\left[\omega_{1},\eta ,C_{0},C_{1},C_{2},\delta \right]$ in $L_{H_{6}}$, B calculates $m^{\prime }\|k^{\prime }=C_{3}⊕\delta$ and calculates $R^{\prime }=H_{5}\left(m^{\prime },k^{\prime }\right)$. If $C_{0}=g^{R^{\prime }}$ and there exists a tuple $\left[\omega_{2},\pi \right]$ in $L_{H_{8}}$ such that $C_{4}=H_{7}{\left(m^{\prime }\right)}^{R^{\prime }}\cdot \pi$ holds, it outputs $m^{\prime }$. When $L_{H_{8}}$ has no such tuple, B outputs ⊥.
  - $O_{Auth}\left(snd,\rho_{j}\right)$: Let $snd=\sigma_{i}$. B performs a simulation algorithm to query $O_{H_{3}}\left(\rho_{j}\right)$ and $O_{H_{1}}\left(\sigma_{i}\right)$.
    - When $j\neq j^{*}$, B can query $O_{RKGen}\left(\rho_{j}\right)$ to obtain $dk_{\rho_{j}}$ and returns $td_{\left(\sigma_{i},\rho_{j}\right)}=Auth\left(pp,\sigma_{i},dk_{\rho_{j}}\right)$.
    - Otherwise, there are a tuple $\left[\rho_{j^{*}},v_{j^{*}}\right]$ in $L_{H_{2}}$ and a tuple $\left[\rho_{j^{*}},t_{j^{*}}\right]$ in $L_{H_{3}}$, and B can query $O_{SKGen}\left(\sigma_{i}\right)$ to obtain $ek_{\sigma_{i}}$, calculates $\eta =e(ek_{\sigma_{i}},H_{3}\left(\rho_{j}\right))$, I=H(η), $\Omega =H_{4}\left(\eta \right)$, and $d_{3}=H_{3}{\left(\rho_{j^{*}}\right)}^{a}={\hat{g}}^{av_{j^{*}}}$, randomly selects $y\in Z_{q}^{*}$, and returns $td_{\left(\sigma_{i},\rho_{j^{*}}\right)}=$$\left(y_{1},y_{2}\right)=\left(d_{3}^{\Omega }{\left(\hat{f}{\hat{h}}^{I}\right)}^{y},{\hat{g}}^{y}\right)$.
(3) *Challenge*: $A_{2}$ submits a pair of sender/receiver identities ($\sigma^{*},{rcv}^{*}$) to B. Let ${rcv}^{*}=\rho^{*}$. Afterwards, B chooses a message $m^{*}\in {\left\{0,1\right\}}^{\lambda }$ randomly and executes a simulation algorithm to query $O_{H_{1}}\left(\sigma^{*}\right)$ and $O_{H_{3}}\left(\rho^{*}\right)$.
  - When the $i^{*}$-th tuple in $L_{H_{1}}$ is $\left[\sigma^{*},u^{*}\right]$ and the $j^{*}$-th tuple in $L_{H_{2}}$ is $\left[\rho^{*},v^{*}\right]$, B randomly selects $k\in {\left\{0,1\right\}}^{l}$, $C_{3}^{*}\in {\left\{0,1\right\}}^{\lambda +l}$, calculates $ek_{\sigma^{*}}=g^{su^{*}}$, $\eta^{*}=e{\left(g,\hat{g}\right)}^{bsu^{*}t^{*}}$, $R=H_{5}\left(m_{x},k\right)$, $C_{0}^{*}=g^{R}$, $C_{1}^{*}=g^{c}$, $C_{2}^{*}=g^{\beta_{0}^{\prime }c}$, and $C_{4}^{*}={H_{7}\left(m^{*}\right)}^{R}\cdot H_{8}\left(e(g^{c},{\hat{g}}^{at^{*}\cdot H_{4}\left(\eta^{*}\right)})\right)$, and delivers this to $A_{2}$ with the challenge ciphertext $C^{*}=\left(C_{0}^{*},C_{1}^{*},C_{2}^{*},C_{3}^{*},C_{4}^{*}\right)$.
    The above construction implicitly sets $\omega_{1}^{*}=e{\left(g,\hat{g}\right)}^{abcv^{*}\cdot H_{4}\left(\eta^{*}\right)}$, $H_{6}\left(\omega_{1}^{*},\eta^{*},C_{0}^{*},C_{1}^{*},C_{2}^{*}\right)$$=\left(m^{*}\|k\right)⊕C_{3}^{*}$, where $g^{u^{*}}=H_{1}\left(\sigma^{*}\right)$, ${\hat{g}}^{bv^{*}}=H_{2}\left(\rho^{*}\right)$, ${\hat{g}}^{t^{*}}=H_{3}\left(\rho^{*}\right)$.
  - Otherwise, B is aborted by failure.
(4) *Phase2*: $A_{2}$ makes issues like in *Phase1*.
(5) *Guess*: $A_{2}$ answers a guess $m^{\prime }$. B randomly chooses a tuple $\left[\omega_{1}^{*},\eta^{*},C_{0}^{*},C_{1}^{*},C_{2}^{*},\delta^{*}\right]$ from $L_{H_{6}}$ and answers the BDH instance solution ${\omega_{1}^{*}}^{{\left(v^{*}H_{4}\left(\eta^{*}\right)\right)}^{-1}}\left(=e{\left(g,\hat{g}\right)}^{abc}\right)$.

□

*Analysis*: It is obvious that the simulations of $O_{H}$, $O_{H_{1}}$, $O_{H_{2}}$, $O_{H_{3}}$, $O_{H_{5}}$, $O_{H_{7}}$, and $O_{H_{8}}$ are perfect. Denote the query $O_{H_{6}}\left(e{\left(g,\hat{g}\right)}^{abcv^{*}\cdot H_{4}\left(\eta^{*}\right)},\eta^{*},C_{0}^{*},C_{1}^{*},C_{2}^{*}\right)$ as the event $AskH_{6}^{*}$. Denote the failure of B to decrypt the legitimate ciphertext in $O_{Dec}$ as the event Derr. Hence, we have, $\mathrm{Pr}\left[Derr\right]\leq \frac{q_{D}}{2^{\lambda +l}}$. Let ${rcv}^{*}=\rho^{*}$. Suppose AbortRK as the event in which B terminates upon the query $O_{RKGen}\left(\rho^{*}\right)$ being issued and AbortCh the event in which B terminates in the challenge phase. Clearly, ¬AbortCh implies ¬AbortRK, because the query $O_{RKGen}\left(\rho^{*}\right)$ cannot be issued. We obtain $\mathrm{Pr}\left[\neg AbortCh\right]\geq \frac{1}{q_{H_{1}}q_{H_{2}}}$.

Define $E=(AskH_{6}^{*}\vee Derr)|\neg AbortCh$. There is no greater over $\frac{1}{2^{\lambda }}$ advantage that $A_{2}$ will gain in guessing *m* when E does not happen, because $O_{H_{6}}$ is a random oracle. $\mathrm{Pr}\left[m=m^{\prime }|\neg E\right]\leq \frac{1}{2^{\lambda }}$. Hence,

$$\begin{array}{cc} \mathrm{Pr}[m=m^{\prime }] & =\mathrm{Pr}\left[m=m^{\prime }|\neg E\right]\mathrm{Pr}\left[\neg E\right]+\mathrm{Pr}\left[m=m^{\prime }|E\right]\mathrm{Pr}\left[E\right]\\ \; & \leq \frac{1}{2^{\lambda }}\mathrm{Pr}\left[\neg E\right]+\mathrm{Pr}\left[E\right]=\frac{1}{2^{\lambda }}+\frac{1}{2}\mathrm{Pr}\left[E\right]\\ \; & =\left(1-\frac{1}{2^{\lambda }}\right)\mathrm{Pr}\left[E\right]+\frac{1}{2^{\lambda }}. \end{array}$$

With ϵ, we obtain

$$\begin{array}{cc} \epsilon & =|\mathrm{Pr}\left[m=m^{\prime }\right]|\leq \left(1-\frac{1}{2^{\lambda }}\right)\mathrm{Pr}\left[E\right]+\frac{1}{2^{\lambda }}\leq \left(1-\frac{1}{2^{\lambda }}\right)\frac{\mathrm{Pr}\left[AskH_{6}^{*}\right]+\mathrm{Pr}\left[Derr\right]}{\mathrm{Pr}[\neg AbortCh]}+\frac{1}{2^{\lambda }}. \end{array}$$

Subsequently, we obtain

$$\begin{array}{cc} \mathrm{Pr}[AskH_{6}^{*}] & \geq \frac{\epsilon -\frac{1}{2^{\lambda }}}{1-\frac{1}{2^{\lambda }}}\mathrm{Pr}\left[\neg AbortCh\right]-\mathrm{Pr}\left[Derr\right]\geq \frac{\epsilon -\frac{1}{2^{\lambda }}}{q_{H_{1}}q_{H_{2}}}-\frac{q_{D}}{2^{\lambda +l}}. \end{array}$$

When $AskH_{6}^{*}$ happens, $A_{2}$ can distinguish the simulation of the challenge ciphertext $C^{*}$. Because $\left[e{\left(g,\hat{g}\right)}^{abcv^{*}\cdot H_{4}\left(\eta^{*}\right)},\eta^{*},C_{0}^{*},C_{1}^{*},C_{2}^{*},\delta^{*}\right]$ has been documented in $L_{H_{6}}$ with non-negligible probability, B is winning when the right element is selected from $L_{H_{6}}$. Thus, the BDH assumption can be addressed by B with advantage $\epsilon^{\prime }\geq \frac{1}{q_{H_{6}}}\mathrm{Pr}\left[AskH_{6}^{*}\right]\geq \frac{1}{q_{H_{6}}}\left(\frac{\epsilon -\frac{1}{2^{\lambda }}}{q_{H_{1}}q_{H_{2}}}-\frac{q_{D}}{2^{\lambda +l}}\right).$

**Theorem** **3.** *For any $A_{3}$, our IBME-ET scheme meets ANON-ID-CCA security on the basis of the Gap-BDH assumption.*

*More precisely, if $A_{3}$ is able to break our proposal with the advantage ϵ, we are able to conceive of a PPT algorithm B to address the Gap-BDH assumption with the advantage $\epsilon^{\prime }\geq \frac{\epsilon }{q_{H_{1}}^{2}q_{H_{2}}^{2}}-\frac{q_{D}}{2^{\lambda +l}},$ where $q_{H_{i}}\left(i=1,2\right)$ and $q_{D}$ denote the numbers of different queries to $O_{H_{i}}\left(i=1,2\right)$ and $O_{Dec}$, respectively.*

**Proof.**  Given a Gap-BDH assumption instance $\left(g,g^{a},g^{c},\hat{g},{\hat{g}}^{a},{\hat{g}}^{b},O_{\mathrm{DBDH}}\right)$, the task of B is to calculate $e{\left(g,\hat{g}\right)}^{abc}$ by interacting with $A_{3}$ as below:

(1) *Setup*: B randomly selects $i_{0}^{*},i_{1}^{*}\in \left\{1,2,\cdots ,q_{H_{1}}\right\}$ and $j_{0}^{*},j_{1}^{*}\in \left\{1,2,\cdots ,q_{H_{2}}\right\}$. B randomly selects $\alpha^{\prime },\beta_{0},\beta_{1}\in Z_{q}^{*}$, calculates $g_{1}=g^{a\alpha^{\prime }}$, $f=g^{\beta_{0}}$, $h=g^{a\beta_{1}}$, $\hat{f}={\hat{g}}^{\beta_{0}}$ and $\hat{h}={\hat{g}}^{a\beta_{1}}$, sets $pp=(G,g,\hat{g},g_{1},f,h,\hat{f},\hat{h},H,H_{i}\left(i=1,2,3,4,5,6,7,8\right))$, and delivers this to $A_{3}$ with pp. B implicitly sets $mk=\left(s,\alpha \right)=\left(a,a\alpha^{\prime }\right)$, because B has no knowledge about *a*. B preserves the $L_{H}$, $L_{H_{i}}\left(i=1,2,3,4,5,6,7,8\right)$, and $L_{A}$ lists to simulate $O_{H}$, $O_{H_{i}}\left(i=1,2,3,4,5,6,7,8\right)$, and $O_{Auth}$. Afterwards, B randomly selects $u_{i_{0}^{*}},u_{i_{1}^{*}},v_{j_{0}^{*}},v_{j_{1}^{*}},t_{j_{0}^{*}},t_{j_{1}^{*}}\in Z_{q}^{*}$ and randomly chooses ${\tilde{\Omega }}_{00},{\tilde{\Omega }}_{01},{\tilde{\Omega }}_{11},{\tilde{\Omega }}_{10},I_{00},\;I_{01},I_{11},I_{10}\in Z_{q}^{*}$.
(2) *Phase1*: B answers $A_{3}$’s queries.
  - $O_{H}\left(\eta \right)$: B executes the following operations.
    - When $O_{\mathrm{DBDH}}(g,g^{a},g^{c},\hat{g},$ ${\hat{g}}^{a},{\hat{g}}^{b},\eta^{{\left(u_{i_{0}^{*}}t_{j_{0}^{*}}\right)}^{-1}})=1$, B returns the Gap-BDH instance solution $\eta^{{\left(u_{i_{0}^{*}}t_{j_{0}^{*}}\right)}^{-1}}\;\left(=e{\left(g,\hat{g}\right)}^{abc}\right)$ and defines $\Omega =\frac{{\tilde{\Omega }}_{00}}{t_{j_{0}^{*}}}$ and $I=I_{00}$.
    - When $O_{\mathrm{DBDH}}(g,g^{a},g^{c},\hat{g},$ ${\hat{g}}^{a},{\hat{g}}^{b},\eta^{{\left(u_{i_{1}^{*}}t_{j_{1}^{*}}\right)}^{-1}})=1$, B returns the Gap-BDH instance solution $\eta^{{\left(u_{i_{1}^{*}}t_{j_{1}^{*}}\right)}^{-1}}\;\left(=e{\left(g,\hat{g}\right)}^{abc}\right)$ and defines $\Omega =\frac{{\tilde{\Omega }}_{11}}{t_{j_{1}^{*}}}$ and $I=I_{11}$.
    - When $O_{\mathrm{DBDH}}(g,g^{a},g^{c},\hat{g},$ ${\hat{g}}^{a},{\hat{g}}^{b},\eta^{{\left(u_{i_{0}^{*}}t_{j_{1}^{*}}\right)}^{-1}})=1$, B returns the Gap-BDH instance solution $\eta^{{\left(u_{i_{0}^{*}}t_{j_{1}^{*}}\right)}^{-1}}\;\left(=e{\left(g,\hat{g}\right)}^{abc}\right)$ and defines $\Omega =\frac{{\tilde{\Omega }}_{01}}{t_{j_{1}^{*}}}$ and $I=I_{01}$.
    - When $O_{\mathrm{DBDH}}(g,g^{a},g^{c},\hat{g},$ ${\hat{g}}^{a},{\hat{g}}^{b},\eta^{{\left(u_{i_{1}^{*}}t_{j_{0}^{*}}\right)}^{-1}})=1$, B returns the Gap-BDH instance solution $\eta^{{\left(u_{i_{1}^{*}}t_{j_{0}^{*}}\right)}^{-1}}\;\left(=e{\left(g,\hat{g}\right)}^{abc}\right)$ and defines $\Omega =\frac{{\tilde{\Omega }}_{10}}{t_{j_{0}^{*}}}$ and $I=I_{10}$.
    - Otherwise, B randomly selects $I,\Omega \in Z_{q}^{*}$.
    Subsequently, B inserts [η,Ω] into $L_{H_{4}}$ and [η,I] into $L_{H}$ and answers *I*.
  - $O_{H_{1}}\left(\sigma_{i}\right)$: Suppose $\sigma_{i}$ as the *i*-th different query. When $i=i_{0}^{*}$, B inserts a tuple $\left[\sigma_{i_{0}^{*}},u_{i_{0}^{*}}\right]$ into $L_{H_{1}}$ and returns $g^{cu_{i_{0}^{*}}}$. When $i=i_{1}^{*}$, B inserts a tuple $\left[\sigma_{i_{1}^{*}},u_{i_{1}^{*}}\right]$ into $L_{H_{1}}$ and returns $g^{cu_{i_{1}^{*}}}$. Otherwise, B randomly selects $u_{i}\in Z_{q}^{*}$, inserts a tuple $\left[\sigma_{i},u_{i}\right]$ into $L_{H_{1}}$, and returns $g^{u_{i}}$.
  - $O_{H_{2}}\left(\rho_{j}\right)$: Suppose $\rho_{j}$ as the *j*-th different query. When $j=j_{0}^{*}$, B inserts a tuple $\left[\rho_{j_{0}^{*}},v_{j_{0}^{*}}\right]$ into $L_{H_{2}}$ and returns ${\hat{g}}^{bv_{j_{0}^{*}}}$. When $j=j_{1}^{*}$, B inserts a tuple $\left[\rho_{j_{1}^{*}},v_{j_{1}^{*}}\right]$ into $L_{H_{2}}$ and returns ${\hat{g}}^{bv_{j_{1}^{*}}}$. Otherwise, B randomly selects $v_{j}\in Z_{q}^{*}$, inserts a tuple $\left[\rho_{j},v_{j}\right]$ into $L_{H_{2}}$, and returns ${\hat{g}}^{v_{j}}$.
  - $O_{H_{3}}\left(\rho_{j}\right)$: B performs a simulation algorithm to query $O_{H_{2}}\left(\rho_{j}\right)$. Subsequently, B searches the tuple $\left[\rho_{j},v_{j}\right]$ in $L_{H_{2}}$. When $j=j_{0}^{*}$, B inserts a tuple $\left[\rho_{j_{0}^{*}},t_{j_{0}^{*}}\right]$ into $L_{H_{3}}$ and returns ${\hat{g}}^{bt_{j_{0}^{*}}}$. When $j=j_{1}^{*}$, B inserts a tuple $\left[\rho_{j_{1}^{*}},t_{j_{1}^{*}}\right]$ into $L_{H_{3}}$ and returns ${\hat{g}}^{bt_{j_{1}^{*}}}$. Otherwise, B randomly selects $t_{j}\in Z_{q}^{*}$, inserts a tuple $\left[\rho_{j},t_{j}\right]$ into $L_{H_{3}}$, and returns ${\hat{g}}^{t_{j}}$.
  - $O_{H_{4}}\left(\eta \right)$: B performs a simulation algorithm to query $O_{H}\left(\eta \right)$. Subsequently, B searches for the tuple [η,Ω] in $L_{H_{4}}$ and returns Ω.
  - $O_{H_{5}}\left(m,k\right)$: B randomly chooses $R\in Z_{q}^{*}$, inserts a tuple [m,k,R] into $L_{H_{5}}$, and answers *R*.
  - $O_{H_{6}}\left(\omega_{1},\eta ,C_{0},C_{1},C_{2}\right)$: B performs a simulation algorithm to query $O_{H}\left(\eta \right)$. Subsequently, B randomly selects $\delta \in {\left\{0,1\right\}}^{\lambda +l}$, inserts a tuple $\left[\omega_{1},-,\eta ,C_{0},C_{1},C_{2},\delta \right]$ into $L_{H_{6}}$, and returns δ.
  - $O_{H_{7}}\left(m\right)$: B randomly selects $h_{7}\in \hat{G}$, inserts a tuple $\left[m,h_{7}\right]$ into $L_{H_{7}}$, and returns $h_{7}$.
  - $O_{H_{8}}\left(\omega_{2}\right)$: B randomly selects $\pi \in \hat{G}$, inserts a tuple $\left[\omega_{2},\pi \right]$ into $L_{H_{8}}$, and returns π.
  - $O_{SKGen}\left(\sigma_{i}\right)$: B performs a simulation algorithm to query $O_{H_{1}}\left(\sigma_{i}\right)$. There is a tuple $\left[\sigma_{i},u_{i}\right]$ in $L_{H_{1}}$. When $i\neq i_{0}^{*}$ and $i\neq i_{1}^{*}$, B answers $ek_{\sigma_{i}}=g^{au_{i}}$. Otherwise, B is aborted by failure.
  - $O_{RKGen}\left(\rho_{j}\right)$: B performs a simulation algorithm to query $O_{H_{3}}\left(\rho_{j}\right)$. There are a tuple $\left[\rho_{j},v_{j}\right]$ in $L_{H_{2}}$ and a tuple $\left[\rho_{j},t_{j}\right]$ in $L_{H_{3}}$. When $j\neq j_{0}^{*}$ and $j\neq j_{1}^{*}$, B answers $dk_{\rho_{j}}=\left(d_{1},d_{2},d_{3}\right)=\left({\hat{g}}^{at_{j}},\;{\hat{g}}^{a\alpha^{\prime }v_{j}},\;{\hat{g}}^{a\alpha^{\prime }t_{j}}\right)$. Otherwise, B is aborted by failure.
  - $O_{Enc}\left(\sigma_{i},rcv,m\right)$: Let $rcv=\rho_{j}$. B performs a simulation algorithm to query $O_{H_{1}}\left(\sigma_{i}\right)$ and $O_{H_{3}}\left(\rho_{j}\right)$. When $i\neq i_{0}^{*}$ and $i\neq i_{1}^{*}$, B can query $O_{SKGen}\left(\sigma_{i}\right)$ to obtain $ek_{\sigma_{i}}$ and returns $C=Enc(pp,ek_{\sigma_{i}},\rho_{j},$m). Otherwise, B executes as below:
    - When $\left(i,j\right)=\left(i_{0}^{*},j_{0}^{*}\right)$ or $\left(i,j\right)=\left(i_{1}^{*},j_{1}^{*}\right)$, B is aborted by failure.
    - When $\left(i,j\right)=\left(i_{0}^{*},j_{1}^{*}\right)$ or $\left(i,j\right)=\left(i_{1}^{*},j_{0}^{*}\right)$, B executes a simulation algorithm to query $O_{Auth}\left(\sigma_{i},\rho_{j}\right)$. There is a tuple $[\sigma_{i},\rho_{j},I,\;\Omega ,$${td}_{\left(\sigma_{i},\rho_{j}\right)}]$ in $L_{A}$. Afterwards, B randomly selects $r\in Z_{q}^{*}$, $\delta \in {\left\{0,1\right\}}^{\lambda +l}$, $k\in {\left\{0,1\right\}}^{l}$, calculates $\omega_{1}=e(g_{1},H_{2}{\left(\rho_{j})\right)}^{r\cdot \Omega }$, $\omega_{2}=e(g_{1},H_{3}{\left(\rho_{j})\right)}^{r\cdot \Omega }$ and $R=H_{5}\left(m,k\right)$, inserts a tuple $\left[\omega_{1},\left(i,j\right),-,C_{0},C_{1},C_{2},\delta \right]$ into $L_{H_{6}}$, and returns $C=(C_{0},C_{1},C_{2},C_{3},C_{4})$, where $C_{0}=g^{R}$, $C_{1}=g^{r}$, $C_{2}={\left(fh^{I}\right)}^{r}$, $C_{3}=\left(m\|k\right)⊕\delta$, $C_{4}=H_{7}{\left(m\right)}^{R}\cdot H_{8}\left(\omega_{2}\right)$.
    - Otherwise, B can query $O_{RKGen}\left(\rho_{j}\right)$ to get $dk_{\rho_{j}}=\left(d_{1},d_{2},d_{3}\right)$, selects $k\in {\left\{0,1\right\}}^{l}$, $r\in Z_{q}^{*}$ randomly, calculates $\eta =e(H_{1}\left(\sigma_{i}\right),d_{1})$, $\omega_{1}=e(g_{1},H_{2}{\left(\rho_{j})\right)}^{r\cdot H_{4}\left(\eta \right)}$, $\omega_{2}=e(g_{1},H_{3}{\left(\rho_{j})\right)}^{r\cdot H_{4}\left(\eta \right)}$ and $R=H_{5}\left(m,k\right)$, and returns $C=(C_{0},C_{1},C_{2},C_{3},C_{4})$, where $C_{0}=g^{R}$, $C_{1}=g^{r}$, $C_{2}={\left(fh^{H(\eta )}\right)}^{r}$, $C_{3}=\left(m\|k\right)⊕H_{6}\left(\omega_{1},\eta ,C_{0},C_{1},C_{2}\right)$, $C_{4}=H_{7}{\left(m\right)}^{R}\cdot H_{8}\left(\omega_{2}\right)$.
  - $O_{Dec}\left(\rho_{j},snd,C\right)$: Let $snd=\sigma_{i}$. B performs a simulation algorithm to query $O_{H_{2}}\left(\rho_{j}\right)$ and $O_{H_{1}}\left(\sigma_{i}\right)$. When $j\neq j_{0}^{*}$ and $j\neq j_{1}^{*}$, B can query $O_{RKGen}\left(\rho_{j}\right)$ to obtain $dk_{\rho_{j}}$ and returns the outcome of $Dec(pp,dk_{\left(\rho_{j}\right)},\sigma_{i},C)$. Otherwise, B executes the following operations:
    - When $\left(i,j\right)=\left(i_{0}^{*},j_{0}^{*}\right)$, or $\left(i,j\right)=\left(i_{1}^{*},j_{1}^{*}\right)$m or $\left(i,j\right)=\left(i_{0}^{*},j_{1}^{*}\right)$, or $\left(i,j\right)=\left(i_{1}^{*},j_{0}^{*}\right)$, B searches for the tuple $[\sigma_{i},\rho_{j},I,\Omega ,$${td}_{\left(\sigma_{i},\rho_{j}\right)}]$ in $L_{A}$. When $L_{A}$ has no such tuple, B executes as below.
      When $\left(i,j\right)=\left(i_{0}^{*},j_{0}^{*}\right)$, $\Omega =\frac{{\tilde{\Omega }}_{00}}{t_{j}}$, $I=I_{00}$. When $\left(i,j\right)=\left(i_{1}^{*},j_{1}^{*}\right)$, $\Omega =\frac{{\tilde{\Omega }}_{11}}{t_{j}}$, $I=I_{11}$. When $\left(i,j\right)=\left(i_{0}^{*},j_{1}^{*}\right)$, $\Omega =\frac{{\tilde{\Omega }}_{01}}{t_{j}}$, $I=I_{01}$. When $\left(i,j\right)=\left(i_{1}^{*},j_{0}^{*}\right)$, $\Omega =\frac{{\tilde{\Omega }}_{10}}{t_{j}}$, $I=I_{10}$. Afterwards, B randomly selects $\tilde{y}\in Z_{q}^{*}$, calculates $z=\frac{t_{j}\alpha^{\prime }\Omega }{\beta_{1}I}$, implicitly sets $y=\tilde{y}-bz$, sets $td_{\left(\sigma_{i},\rho_{j}\right)}=\left(y_{1},y_{2}\right)=\left({\hat{g}}^{\beta_{0}\left(\tilde{y}-bz\right)}{\hat{g}}^{a\beta_{1}I\tilde{y}},\;{\hat{g}}^{\tilde{y}-bz}\right)$, and stores $\left[\rho_{j},\sigma_{i},I,\Omega ,{td}_{\left(\sigma_{i},\rho_{j}\right)}\right]$ in $L_{A}$. $td_{\left(\sigma_{i},\rho_{j}\right)}=\left(y_{1},y_{2}\right)$ is a valid random trapdoor according to $\rho_{j}$ and $\sigma_{i}$, where
      $$\begin{array}{cc} y_{1} & ={\hat{g}}^{\beta_{0}\left(\tilde{y}-bz\right)}{\hat{g}}^{a\beta_{1}I\tilde{y}}={\hat{g}}^{a\alpha^{\prime }bt_{j}\cdot \Omega }{\hat{g}}^{\beta_{0}y}{\hat{g}}^{a\beta_{1}Iy}=d_{3}^{\Omega }{\hat{g}}^{(\beta_{0}+a\beta_{1}I)y}=d_{3}^{\Omega }{\left(\hat{f}{\hat{h}}^{I}\right)}^{y},\\ \;y_{2} & ={\hat{g}}^{\tilde{y}-bz}={\hat{g}}^{y}. \end{array}\;$$
      Next, B calculates $\omega_{2}=\frac{e(C_{1},y_{1})}{e(C_{2},y_{2})}$. For each tuple $[\omega_{1},\left(i,j\right),-,$$C_{0},C_{1},C_{2},\delta ]$ in $L_{H_{6}}$, B calculates $m\|k=C_{3}⊕\delta$ and $R=H_{5}\left(m,k\right)$. If both $C_{0}=g^{R}$ and $C_{4}=H_{7}{\left(m\right)}^{R}\cdot H_{8}\left(\omega_{2}\right)$ hold, B returns *m*; otherwise, B returns ⊥.
    - Otherwise, B can query $O_{Auth}\left(\sigma_{i},\rho_{j}\right)$ to obtain $td_{\left(\sigma_{i},\rho_{j}\right)}=\left(y_{1},y_{2}\right)$ and calculates $\omega_{2}=\frac{e(C_{1},y_{1})}{e(C_{2},y_{2})}$. For each tuple $[\omega_{1},\left(i,j\right),-,$$C_{0},C_{1},C_{2},\delta ]$ in $L_{H_{6}}$, B calculates $m\|k=C_{3}⊕\delta$ and $R=H_{5}\left(m,k\right)$. If both $C_{0}=g^{R}$ and $C_{4}=H_{7}{\left(m\right)}^{R}\cdot H_{8}\left(\omega_{2}\right)$ hold, B returns *m*; otherwise, B returns ⊥.
  - $O_{Auth}\left(snd,\rho_{j}\right)$: Let $snd=\sigma_{i}$. B performs a simulation algorithm to query $O_{H_{3}}\left(\rho_{j}\right)$ and $O_{H_{1}}\left(\sigma_{i}\right)$. There is a tuple $\left[\rho_{j},v_{j}\right]$ in $L_{H_{2}}$. When $j\neq j_{0}^{*}$ and $j\neq j_{1}^{*}$, B can query $O_{RKGen}\left(\rho_{j}\right)$ to obtain $dk_{\rho_{j}}=\left(d_{1},d_{2},d_{3}\right)$, calculates $\eta =e(H_{1}\left(\sigma_{i}\right),d_{1})$, I=H(η), $\Omega =H_{3}\left(\eta \right)$, returns $td_{\left(\sigma_{i},\rho_{j}\right)}=Auth\left(pp,\sigma_{i},dk_{\rho_{j}}\right)$, and stores $\left[\sigma_{i},\rho_{j},I,\Omega ,td_{\left(\sigma_{i},\rho_{j}\right)}\right]$ into $L_{A}$. Otherwise, B executes as below:
    - When $\left(i,j\right)=\left(i_{0}^{*},j_{0}^{*}\right)$ or $\left(i,j\right)=\left(i_{1}^{*},j_{1}^{*}\right)$, B is aborted by failure.
    - When $\left(i,j\right)=\left(i_{0}^{*},j_{1}^{*}\right)$, $\Omega =\frac{{\tilde{\Omega }}_{01}}{t_{j}}$, $I=I_{01}$.
    - When $\left(i,j\right)=\left(i_{1}^{*},j_{0}^{*}\right)$, $\Omega =\frac{{\tilde{\Omega }}_{10}}{t_{j}}$, $I=I_{10}$.
    - Otherwise, B can query $O_{SKGen}\left(\sigma_{i}\right)$ to obtain $ek_{\sigma_{i}}$ and calculates $\eta =e(ek_{\sigma_{i}},$$H_{3}\left(\rho_{j}\right))$, $\Omega =H_{4}\left(\eta \right)$. I=H(η)
    Subsequently, B randomly selects $\tilde{y}\in Z_{q}^{*}$, calculates $z=\frac{t_{j}\alpha^{\prime }\Omega }{I\beta_{1}}$, implicitly sets $y=\tilde{y}-bz$, returns $td_{\left(\sigma_{i},\rho_{j}\right)}=\left(y_{1},y_{2}\right)=\left({\hat{g}}^{\beta_{0}\left(\tilde{y}-bz\right)}{\hat{g}}^{a\beta_{1}I\tilde{y}},\;{\hat{g}}^{\tilde{y}-bz}\right)$, and then, stores $[\sigma_{i},\rho_{j},I,$$\Omega ,{td}_{\left(\sigma_{i},\rho_{j}\right)}]$ in $L_{A}$. ${td}_{\left(\sigma_{i},\rho_{j}\right)}=\left(y_{1},y_{2}\right)$ is a valid random trapdoor according to $\rho_{j}$ and $\sigma_{i}$, where
    $$\begin{array}{cc} y_{1} & ={\hat{g}}^{\beta_{0}\left(\tilde{y}-bz\right)}{\hat{g}}^{a\beta_{1}I\tilde{y}}={\hat{g}}^{a\alpha^{\prime }bt_{j}\cdot \Omega }{\hat{g}}^{\beta_{0}y}{\hat{g}}^{a\beta_{1}Iy}=d_{3}^{\Omega }{\hat{g}}^{(\beta_{0}+a\beta_{1}I)y}=d_{3}^{\Omega }{\left(\hat{f}{\hat{h}}^{I}\right)}^{y},\\ \;y_{2} & ={\hat{g}}^{\tilde{y}-bz}={\hat{g}}^{y}. \end{array}$$
(3) *Challenge*: $A_{3}$ offers a message $m^{*}\in {\left\{0,1\right\}}^{\lambda }$ and two pairs of sender/receiver identities (${snd}_{0}^{*},\rho_{0}^{*}$), (${snd}_{1}^{*},\rho_{1}^{*}$) to B. Set ${snd}_{0}^{*}=\sigma_{0}^{*}$, ${snd}_{1}^{*}=\sigma_{1}^{*}$. Afterwards, B utilizes a simulation algorithm to query $O_{H_{1}}\left(\sigma_{0}^{*}\right)$, $O_{H_{1}}\left(\sigma_{1}^{*}\right)$, $O_{H_{3}}\left(\rho_{0}^{*}\right)$, and $O_{H_{3}}\left(\rho_{1}^{*}\right)$:
  - When the $i_{0}^{*}$-th tuple in $L_{H_{1}}$ is $\left[\sigma_{0}^{*},u_{0}^{*}\right]$, the $i_{1}^{*}$-th tuple in $L_{H_{1}}$ is $\left[\sigma_{1}^{*},u_{1}^{*}\right]$, the $j_{0}^{*}$-th tuple in $L_{H_{2}}$ is $\left[\rho_{0}^{*},v_{0}^{*}\right]$, and the $j_{1}^{*}$-th tuple in $L_{H_{2}}$ is $\left[\rho_{1}^{*},v_{1}^{*}\right]$, B executes the following operations:
    Firstly, B randomly selects x∈{0,1} and searches for the tuple $\left[\sigma_{x}^{*},\rho_{x}^{*},I,\Omega ,td_{\left(\sigma_{x}^{*},\rho_{x}^{*}\right)}\right]$ in $L_{A}$. When $L_{A}$ has no such tuple, B sets $\Omega =\frac{{\tilde{\Omega }}_{xx}}{t_{x}^{*}}$ and $I=I_{xx}$. Subsequently, B randomly selects $\tilde{y}\in Z_{q}^{*}$, calculates $z=\frac{t_{x}\alpha^{\prime }\Omega }{\beta_{1}I}=\frac{\alpha^{\prime }{\tilde{\Omega }}_{xx}}{\beta_{1}I}$, implicitly sets $y=\tilde{y}-bz$, obtains $td_{\left(\sigma_{x}^{*},\rho_{x}^{*}\right)}=\left(y_{1},y_{2}\right)=\left({\hat{g}}^{\beta_{0}\left(\tilde{y}-bz\right)}{\hat{g}}^{a\beta_{1}I\tilde{y}},\;{\hat{g}}^{\tilde{y}-bz}\right)$, and then, inserts a tuple $\left[\sigma_{x}^{*},\rho_{x}^{*},I,\Omega ,{td}_{\left(\sigma_{x}^{*},\rho_{x}^{*}\right)}\right]$ in $L_{A}$. $td_{\left(\sigma_{x}^{*},\rho_{x}^{*}\right)}=\left(y_{1},y_{2}\right)$ is a valid random trapdoor according to $\sigma_{x}^{*}$ and $\rho_{x}^{*}$, where
    $$\begin{array}{cc} y_{1} & ={\hat{g}}^{\beta_{0}\left(\tilde{y}-bz\right)}{\hat{g}}^{a\beta_{1}I\tilde{y}}={\hat{g}}^{ab\alpha^{\prime }\cdot {\tilde{\Omega }}_{xx}}{\hat{g}}^{\beta_{0}y}{\hat{g}}^{a\beta_{1}Iy}={\hat{g}}^{a\alpha^{\prime }bt_{x}\cdot \Omega }{\hat{g}}^{\beta_{0}y}{\hat{g}}^{a\beta_{1}Iy}=d_{3}^{\Omega }{\left(\hat{f}{\hat{h}}^{I}\right)}^{y},\\ \;y_{2} & ={\hat{g}}^{\tilde{y}-bz}={\hat{g}}^{y}. \end{array}$$
    Secondly, B randomly selects $r\in Z_{q}^{*}$, $C_{3}^{*}\in {\left\{0,1\right\}}^{\lambda +l}$, $k\in {\left\{0,1\right\}}^{l}$, calculates $\omega_{2}^{*}=e{\left(g^{a\alpha^{\prime }},{\hat{g}}^{b}\right)}^{r{\tilde{\Omega }}_{xx}}$, $R=H_{5}\left(m^{*},k\right)$, $C_{0}^{*}=g^{R}$, $C_{1}^{*}=g^{r}$, $C_{2}^{*}={\left(fh^{I}\right)}^{r}$, and $C_{4}^{*}=H_{7}{\left(m^{*}\right)}^{R}\cdot H_{8}\left(\omega_{2}^{*}\right)$.
    The above construction implicitly sets $C_{3}^{*}=\left(m^{*}\|k\right)⊕H_{6}\left(\omega_{1}^{*},\eta^{*},C_{0}^{*},C_{1}^{*},C_{2}^{*}\right)$, where $\omega_{1}^{*}=e{\left(g^{a\alpha^{\prime }},{\hat{g}}^{b}\right)}^{r\Omega v_{x}^{*}}$, $\eta^{*}=e{\left(g,\hat{g}\right)}^{abcu_{x}^{*}t_{x}^{*}}$. $C_{x}^{*}=\left(C_{0}^{*},C_{1}^{*},C_{2}^{*},C_{3}^{*},C_{4}^{*}\right)$ is the encryption of $m^{*}$ according to $\sigma_{x}^{*}$ and $\rho_{x}^{*}$, where
    $$\begin{array}{cc} \omega_{2}^{*} & =e{\left(g^{a\alpha^{\prime }},{\hat{g}}^{b}\right)}^{r{\tilde{\Omega }}_{xx}}=e{\left(g_{1},{\hat{g}}^{b}\right)}^{rt_{x}^{*}\Omega }=e(g_{1},{\hat{g}}^{bt_{x}^{*}{)}^{r\cdot \Omega }}={e(g_{1},H_{3}\left(\rho_{x}^{*}\right))}^{r\cdot \Omega }. \end{array}$$
    Eventually, B returns the corresponding challenge ciphertext $C_{x}^{*}=\left(C_{0}^{*},C_{1}^{*},C_{2}^{*},C_{3}^{*},C_{4}^{*}\right)$ and challenge trapdoor $td_{\left(\sigma_{x}^{*},\rho_{x}^{*}\right)}=\left(y_{1},y_{2}\right)$ to $A_{3}$.
  - Otherwise, B is aborted by failure.
(4) *Phase2*: $A_{3}$ makes issues like in *Phase1*.
(5) *Guess*: $A_{3}$ answers a guess $x^{\prime }\in \left\{0,1\right\}$.

□

*Analysis*: It is obvious that the simulations of $O_{H_{1}}$, $O_{H_{2}}$, $O_{H_{3}}$, $O_{H_{5}}$, $O_{H_{7}}$, and $O_{H_{8}}$ are perfect. Define $\eta_{\left(0,0\right)}=e{\left(g,\hat{g}\right)}^{abcu_{i_{0}^{*}}t_{j_{0}^{*}}}$, $\eta_{\left(1,1\right)}=e{\left(g,\hat{g}\right)}^{abcu_{i_{1}^{*}}t_{j_{0}^{*}}}$, $\eta_{\left(1,0\right)}=e{\left(g,\hat{g}\right)}^{abcu_{i_{1}^{*}}t_{j_{0}^{*}}}$, $\eta_{\left(0,1\right)}=e{\left(g,\hat{g}\right)}^{abcu_{i_{0}^{*}}t_{j_{1}^{*}}}$. Let ${snd}_{0}^{*}=\sigma_{0}^{*}$ and ${snd}_{1}^{*}=\sigma_{1}^{*}$. Denote the queries $O_{H}\left(\eta_{\left(0,0\right)}\right)$, $O_{H}\left(\eta_{\left(0,1\right)}\right)$, $O_{H}\left(\eta_{\left(1,0\right)}\right)$, and $O_{H}\left(\eta_{\left(1,1\right)}\right)$ as the event AskH. Suppose AbortSK as the event in which B terminates upon the queries $O_{SKGen}\left(\sigma_{0}^{*}\right)$ and $O_{SKGen}\left(\sigma_{1}^{*}\right)$ being issued, AbortRK as the event in which B terminates upon the queries $O_{RKGen}\left(\rho_{0}^{*}\right)$ and $O_{RKGen}\left(\rho_{1}^{*}\right)$ being issued, AbortAuth as the event in which B terminates upon the queries $O_{Auth}\left(\sigma_{0}^{*},\rho_{0}^{*}\right)$ and $O_{Auth}\left(\sigma_{1}^{*},\rho_{1}^{*}\right)$ being issued, AbortEnc as the event in which B terminates upon the queries $O_{Enc}\left(\sigma_{0}^{*},\rho_{0}^{*},*\right)$ and $O_{Enc}\left(\sigma_{1}^{*},\rho_{1}^{*},*\right)$ being issued, and AbortCh as the event in which B terminates in the challenge phase. Clearly, ¬AbortCh implies ¬AbortSK, ¬AbortRK, ¬AbortAuth, and ¬AbortEnc, because the queries $O_{SKGen}\left(\sigma_{0}^{*}\right)$ and $O_{SKGen}\left(\sigma_{1}^{*}\right)$ cannot be issued, the queries $O_{RKGen}\left(\rho_{0}^{*}\right)$ and $O_{RKGen}\left(\rho_{1}^{*}\right)$ are unable to be issued, $\left(\sigma_{0}^{*},\rho_{0}^{*}\right)$ and the queries $O_{Auth}\left(\sigma_{0}^{*},\rho_{0}^{*}\right)$ and $O_{Auth}\left(\sigma_{1}^{*},\rho_{1}^{*}\right)$ are unable to be issued, and the queries $O_{Enc}\left(\sigma_{0}^{*},\rho_{0}^{*},*\right)$ and $O_{Enc}\left(\sigma_{1}^{*},\rho_{1}^{*},*\right)$ are unable to be issued. Thus, we obtain $\mathrm{Pr}\left[\neg AbortCh\right]\geq \frac{1}{q_{H_{1}}^{2}}\cdot \frac{1}{q_{H_{2}}^{2}}$.

Denote the failure of B to decrypt the legitimate ciphertext in $O_{Dec}$ as the event Derr. Thus, $\mathrm{Pr}\left[Derr\right]\leq \frac{q_{D}}{2^{\lambda +l}}$.

Define $E_{0}=\left(AskH\vee Derr\right)|\neg AbortCh$. There is no greater over $\frac{1}{2}$ advantage that $A_{3}$ will gain in guessing *x* when $E_{0}$ does not happen because $O_{H}$, $O_{H_{4}}$, and $O_{H_{6}}$ are random oracles. Hence, $\mathrm{Pr}\left[x=x^{\prime }|\neg E_{0}\right]=\frac{1}{2}$. We obtain

$$\begin{array}{cc} \mathrm{Pr}[x=x^{\prime }] & =\mathrm{Pr}\left[x=x^{\prime }|\neg E_{0}\right]\mathrm{Pr}\left[\neg E_{0}\right]+\mathrm{Pr}\left[x=x^{\prime }|E_{0}\right]\mathrm{Pr}\left[E_{0}\right]\leq \frac{1}{2}\mathrm{Pr}\left[\neg E_{0}\right]+\mathrm{Pr}\left[E_{0}\right]=\frac{1}{2}+\frac{1}{2}\mathrm{Pr}\left[E_{0}\right]. \end{array}$$

With ϵ, we obtain

$$\begin{array}{cc} \epsilon & =|\mathrm{Pr}\left[x=x^{\prime }\right]-\frac{1}{2}|\leq \mathrm{Pr}\left[E_{0}\right]\leq \frac{\mathrm{Pr}[AskH]+\mathrm{Pr}[Derr]}{\mathrm{Pr}[\neg AbortCh]}. \end{array}$$

Subsequently, we obtain

$$\begin{array}{cc} \mathrm{Pr}[AskH] & \geq \epsilon \mathrm{Pr}\left[\neg AbortCh\right]-\mathrm{Pr}\left[Derr\right]\geq \frac{\epsilon }{q_{H_{1}}^{2}q_{H_{2}}^{2}}-\frac{q_{D}}{2^{\lambda +l}}. \end{array}$$

Obviously, when AskH occurs, the Gap-BDH assumption can certainly be addressed by B. B addresses the Gap-BDH assumption with advantage $\epsilon^{\prime }=\mathrm{Pr}\left[B\;success\right]=\mathrm{Pr}\left[AskH\right]\geq \frac{\epsilon }{q_{H_{1}}^{2}q_{H_{2}}^{2}}-\frac{q_{D}}{2^{\lambda +l}}.$

**Theorem** **4.** *For any $A_{4}$, our IBME-ET scheme meets sUF-ID-CMA security on the basis of the Gap-BDH assumption.*

*More precisely, if $A_{4}$ is able to break our proposal with the advantage ϵ, we are able to conceive of a PPT algorithm B to address the Gap-BDH assumption with the advantage $\epsilon^{\prime }\geq \epsilon \left(1-\frac{1}{q}\right)\frac{1}{q_{H_{1}}q_{H_{3}}}-\frac{1+q_{D}}{2^{\lambda }},$ where $q_{H_{i}}\left(i=1,3\right)$ and $q_{D}$ denote the numbers of different queries to $O_{H_{i}}\left(i=1,3\right)$ and $O_{Dec}$, respectively.*

**Proof.**  Given a Gap-BDH assumption instance $\left(g,g^{a},g^{c},\hat{g},{\hat{g}}^{a},{\hat{g}}^{b},O_{\mathrm{DBDH}}\right)$, the task of B is to calculate $e{\left(g,\hat{g}\right)}^{abc}$ by interacting with $A_{4}$ as below:

(1) *Setup*: B randomly chooses $i^{*}\in \left\{1,2,\cdots ,q_{H_{1}}\right\}$, $j^{*}\in \left\{1,2,\cdots ,q_{H_{3}}\right\}$. B randomly selects $\alpha ,\beta_{0},\beta_{1}\in Z_{q}^{*}$, calculates $g_{1}=g^{\alpha }$, $f=g^{\beta_{0}}$, $h=g^{\beta_{1}}$, $\hat{f}={\hat{g}}^{\beta_{0}}$ and $\hat{h}={\hat{g}}^{\beta_{1}}$, sets $pp=(G,g,\hat{g},g_{1},f,h,\hat{f},\hat{h},H,$$H_{i}\left(i=1,2,3,4,5,6,7,8\right))$, and delivers this to $A_{4}$ with pp. B implicitly sets mk=(a,α), because B has no knowledge about *a*. B preserves the $L_{H}$ and $L_{H_{i}}\left(i=1,2,3,4,5,6,7,8\right)$ lists to simulate $O_{H}$ and $O_{H_{i}}\left(i=1,2,3,4,5,6,7,8\right)$. Afterwards, B randomly selects $I^{*},\Omega^{*}\in Z_{q}^{*}$.
(2) *Queries: *B answers $A_{4}$’s queries as below:
  - $O_{H}\left(\eta \right)$: When $O_{\mathrm{DBDH}}\left(g,g^{a},g^{c},\hat{g},{\hat{g}}^{a},{\hat{g}}^{b},\eta \right)\neq 1$, B randomly selects $\Omega ,I\in Z_{q}^{*}$, inserts [η,Ω] into $L_{H_{4}}$ and [η,I] into $L_{H}$, and answers *I*. Otherwise, B answers the Gap-BDH solution $\eta \;(=e{\left(g,\hat{g}\right)}^{abc})$, defines $\Omega =\Omega^{*}$ and $I=I^{*}$, inserts [η,Ω] into $L_{H_{4}}$ and [η,I] into $L_{H}$, and answers *I*.
  - $O_{H_{1}}\left(\sigma_{i}\right)$: Suppose $\sigma_{i}$ as the *i*-th different query. When $i\neq i^{*}$, B randomly selects $u_{i}\in Z_{q}^{*}$, inserts a tuple $\left[\sigma_{i},u_{i}\right]$ into $L_{H_{1}}$, returns $g^{u_{i}}$. Otherwise, B inserts a tuple $\left[\sigma_{i},-\right]$ into $L_{H_{1}}$ and returns $g^{c}$.
  - $O_{H_{2}}\left(\rho_{j}\right)$: B performs a simulation algorithm to query $O_{H_{3}}\left(\rho_{j}\right)$. Subsequently, B randomly selects $v_{j}\in Z_{q}^{*}$, inserts a tuple $\left[\rho_{j},v_{j}\right]$ into $L_{H_{2}}$, and returns ${\hat{g}}^{v_{j}}$.
  - $O_{H_{3}}\left(\rho_{j}\right)$: Suppose $\rho_{j}$ as the *j*-th different query. When $j\neq j^{*}$, B randomly selects $t_{j}\in Z_{q}^{*}$, inserts a tuple $\left[\rho_{j},t_{j}\right]$ into $L_{H_{3}}$, and returns ${\hat{g}}^{t_{j}}$. Otherwise, B inserts a tuple $\left[\rho_{j},-\right]$ into $L_{H_{3}}$ and returns ${\hat{g}}^{b}$.
  - $O_{H_{4}}\left(\eta \right)$: B performs a simulation algorithm to query $O_{H}\left(\eta \right)$. Subsequently, B searches for the tuple [η,Ω] in $L_{H_{4}}$ and answers Ω.
  - $O_{H_{5}}\left(m,k\right)$: B randomly chooses $R\in Z_{q}^{*}$, inserts a tuple [m,k,R] into $L_{H_{5}}$, and answers *R*.
  - $O_{H_{6}}\left(\omega_{1},\eta ,C_{0},C_{1},C_{2}\right)$: B performs a simulation algorithm to query $O_{H}\left(\eta \right)$. Subsequently, B randomly selects $\delta \in {\left\{0,1\right\}}^{\lambda +l}$, inserts a tuple $\left[\omega_{1},-,\eta ,C_{0},C_{1},C_{2},\delta \right]$ into $L_{H_{6}}$, and returns δ.
  - $O_{H_{7}}\left(m\right)$: B randomly selects $h_{7}\in \hat{G}$, inserts a tuple $\left[m,h_{7}\right]$ into $L_{H_{7}}$, and returns $h_{7}$.
  - $O_{H_{8}}\left(\omega_{2}\right)$: B randomly selects $\pi \in \hat{G}$, inserts a tuple $\left[\omega_{2},\pi \right]$ into $L_{H_{8}}$, and returns π.
  - $O_{SKGen}\left(\sigma_{i}\right)$: B performs a simulation algorithm to query $O_{H_{1}}\left(\sigma_{i}\right)$. There is a tuple $\left[\sigma_{i},u_{i}\right]$ in $L_{H_{1}}$. If $i\neq i^{*}$, B returns $ek_{\sigma_{i}}=g^{au_{i}}$. Otherwise, B is aborted by failure.
  - $O_{RKGen}\left(\rho_{j}\right)$: B performs a simulation algorithm to query $O_{H_{2}}\left(\rho_{j}\right)$. There is a tuple $\left[\rho_{j},v_{j}\right]$ in $L_{H_{2}}$. If $j\neq j^{*}$, B returns $dk_{\rho_{j}}=\left(d_{1},d_{2},d_{3}\right)$$=({\hat{g}}^{at_{j}},\;{\hat{g}}^{\alpha v_{j}},\;{\hat{g}}^{\alpha t_{j}})$. Otherwise, B is aborted by failure.
  - $O_{Auth}\left(snd,\rho_{j}\right)$: Let $snd=\sigma_{i}$. B performs a simulation algorithm to query $O_{H_{2}}\left(\rho_{j}\right)$ and $O_{H_{1}}\left(\sigma_{i}\right)$. There is a tuple $\left[\rho_{j},t_{j}\right]$ in $L_{H_{3}}$. When $j\neq j^{*}$, B can query $O_{RKGen}\left(\rho_{j}\right)$ to obtain $dk_{\rho_{j}}=\left(d_{1},d_{2},d_{3}\right)$, calculates $\eta =e(H_{1}\left(\sigma_{i}\right),d_{1})$, $\Omega =H_{4}\left(\eta \right)$ and I=H(η), and answers $td_{\left(\sigma_{i},\rho_{j}\right)}=Auth\left(pp,\sigma_{i},dk_{\rho_{j}}\right)$. Otherwise, B executes the following operations:
    - When $\left(i,j\right)\neq \left(i^{*},j^{*}\right)$, B can query $O_{SKGen}\left(\sigma_{i}\right)$ to obtain $ek_{\sigma_{i}}$, calculates $\eta =e(ek_{\sigma_{i}},H_{2}\left(\rho_{j}\right))$, I=H(η), and $\Omega =H_{4}\left(\eta \right)$, calculates $d_{3}={\hat{g}}^{\alpha t_{j}}$, randomly selects $y\in Z_{q}^{*}$, and returns $td_{\left(\sigma_{i},\rho_{j}\right)}=$$\left(y_{1},y_{2}\right)=\left(d_{3}^{H_{4}\left(\eta \right)}{\left(\hat{f}{\hat{h}}^{H(\eta )}\right)}^{y},\;{\hat{g}}^{y}\right)$.
    - Otherwise, B defines $\Omega =\Omega^{*}$, $I=I^{*}$, calculates $d_{3}={\hat{g}}^{b\alpha }$, randomly selects $y\in Z_{q}^{*}$, and returns $td_{\left(\sigma_{i},\rho_{j}\right)}=\left(y_{1},y_{2}\right)=\left(d_{3}^{\Omega }{\left(\hat{f}{\hat{h}}^{I}\right)}^{y},\;{\hat{g}}^{y}\right)$.
  - $O_{Enc}\left(\sigma_{i},rcv,m\right)$: Let $rcv=\rho_{j}$. B performs a simulation algorithm to query $O_{H_{1}}\left(\sigma_{i}\right)$ and $O_{H_{2}}\left(\rho_{j}\right)$. When $i\neq i^{*}$, B can query $O_{SKGen}\left(\sigma_{i}\right)$ to obtain $ek_{\sigma_{i}}$ and returns $C=Enc(pp,ek_{\sigma_{i}},\rho_{j},m)$. Otherwise, B executes the following operations:
    - When $\left(i,j\right)\neq \left(i^{*},j^{*}\right)$, B can query $O_{RKGen}\left(\rho_{j}\right)$ to obtain $dk_{\rho_{j}}=\left(d_{1},d_{2},d_{3}\right)$, randomly selects $r\in Z_{q}^{*}$, $k\in {\left\{0,1\right\}}^{l}$, calculates $R=H_{5}\left(m,k\right)$, $\eta =e(H_{1}\left(\sigma_{i}\right),d_{1})$, $\Omega =H_{4}\left(\eta \right)$, $\omega_{1}=e(g_{1},H_{2}{\left(\rho_{j})\right)}^{r\cdot \Omega }$ and $\omega_{2}=e(g_{1},H_{3}{\left(\rho_{j})\right)}^{r\cdot \Omega }$, and then, returns $C=(C_{0},C_{1},C_{2},C_{3},C_{4})$, where $C_{0}=g^{R}$, $C_{1}=g^{r}$, $C_{2}={\left(fh^{I}\right)}^{r}$, $C_{3}=\left(m\|k\right)⊕H_{6}\left(\omega_{1}\right)$, $C_{4}=H_{7}{\left(m\right)}^{R}\cdot H_{8}\left(\omega_{2}\right)$.
    - Otherwise, B defines $\Omega =\Omega^{*}$, $I=I^{*}$, randomly picks $r\in Z_{q}^{*}$, $\delta \in {\left\{0,1\right\}}^{\lambda +l}$, $k\in {\left\{0,1\right\}}^{l}$, calculates $R=H_{5}\left(m,k\right)$, $\omega_{1}=e(g_{1},H_{2}{\left(\rho_{j})\right)}^{r\cdot \Omega }$ and $\omega_{2}=e(g_{1},H_{3}{\left(\rho_{j})\right)}^{r\cdot \Omega }$, inserts a tuple $\left[\omega_{1},\left(i,j\right),-,C_{0},C_{1},C_{2},\delta \right]$ into $L_{H_{6}}$, and then, returns $C=(C_{0},C_{1},C_{2},C_{3},C_{4})$, where $C_{0}=g^{R}$, $C_{1}=g^{r}$, $C_{2}={\left(fh^{I}\right)}^{r}$, $C_{3}=\left(m\|k\right)⊕\delta$, and $C_{4}=H_{7}{\left(m\right)}^{R}\cdot H_{8}\left(\omega_{2}\right)$.
  - $O_{Dec}\left(\rho_{j},snd,C\right)$: Let $snd=\sigma_{i}$. B performs a simulation algorithm to query $O_{H_{2}}\left(\rho_{j}\right)$ and $O_{H_{1}}\left(\sigma_{i}\right)$. When $j\neq j^{*}$, B can query $O_{RKGen}\left(\rho_{j}\right)$ to obtain $dk_{\rho_{j}}$ and returns the outcome of the algorithm $Dec(pp,dk_{\rho_{j}},$$\sigma_{i},C)$. Otherwise, B executes the following operations:
    - When $\left(i,j\right)\neq \left(i^{*},j^{*}\right)$, B can query $O_{Auth}\left(\sigma_{i},\rho_{j}\right)$ to obtain ${td}_{\left(\sigma_{i},\rho_{j}\right)}=\left(y_{1},y_{2}\right)$, calculates $\omega_{2}=\frac{e(C_{1},y_{1})}{e(C_{2},y_{2})}$, obtains $ek_{\sigma_{i}}$ by querying $O_{SKGen}\left(\sigma_{i}\right)$, calculates $\eta =e(ek_{\sigma_{i}},H_{3}\left(\rho_{j}\right))$, $\Omega =H_{4}\left(\eta \right)$, $d_{2}=H_{2}{\left(\rho_{j}\right)}^{\alpha }$, $\omega_{1}=e\left(C_{1},d_{2}^{\Omega }\right)$, recovers m‖k by computing $C_{3}⊕H_{6}\left(\omega_{1},\eta ,C_{0},C_{1},C_{2}\right)$, calculates $R=H_{5}\left(m,k\right)$. If $C_{0}=g^{R}$ and $C_{4}=H_{7}{\left(m\right)}^{R}\cdot H_{8}\left(\omega_{2}\right)$ hold, B answers *m*; otherwise, B answers ⊥.
    - Otherwise, B defines $\Omega =\Omega^{*}$, $I=I^{*}$, calculates $\omega_{1}={e(C_{1},{\hat{g}}^{v_{j}})}^{\Omega }$, obtains ${td}_{\left(\sigma_{i},\rho_{j}\right)}=\left(y_{1},y_{2}\right)$ by querying $O_{Auth}\left(\sigma_{i},\rho_{j}\right)$, calculates $\omega_{2}=\frac{e(C_{1},y_{1})}{e(C_{2},y_{2})}$, and searches for the corresponding tuple $[\omega_{1},\left(i,j\right),-,$$C_{0},C_{1},C_{2},\delta ]$ in $L_{H_{6}}$. If there exists no such tuple in $L_{H_{6}}$, B randomly selects $\delta \in {\left\{0,1\right\}}^{\lambda +l}$ and inserts $[\omega_{1},\left(i,j\right),-,$$C_{0},C_{1},C_{2},\delta ]$ into $L_{H_{6}}$. Afterwards, B recovers m‖k by computing $C_{3}⊕\delta$ and calculates $R=H_{5}\left(m,k\right)$. If $C_{0}=g^{R}$ and $C_{4}=H_{7}{\left(m\right)}^{R}\cdot H_{8}\left(\omega_{2}\right)$ hold, B answers *m*; otherwise, B answers ⊥.
(3) *Forgery: *$A_{4}$ outputs a triple $\left(snd^{*},\rho^{*},C^{*}\right)$, where $snd^{*}=\sigma^{*}$ and $C^{*}=\left(C_{0}^{*},C_{1}^{*},C_{2}^{*},C_{3}^{*},C_{4}^{*}\right)$.

□

*Analysis*: It is obvious that the simulations of $O_{H_{1}}$, $O_{H_{2}}$, $O_{H_{3}}$, $O_{H_{5}}$, $O_{H_{7}}$, and $O_{H_{8}}$ are perfect. Define $\eta^{*}=e{\left(g,\hat{g}\right)}^{abc}$. Denote the query $O_{H}\left(\eta^{*}\right)$ as the event AskH. Denote the failure of B to decrypt the legitimate ciphertext in $O_{Dec}$ as the event Derr. Thus, $\mathrm{Pr}\left[Derr\right]\leq \frac{q_{D}}{2^{\lambda +l}}$.

Suppose *E* as the event for which $\sigma^{*}=\sigma_{i^{*}}$, $\rho^{*}=\rho_{j^{*}}$, and $\left(\sigma^{*},\rho^{*},C^{*}\right)$ are legitimate. With ϵ and the lemma on the relationship between the chosen-identity attack and given identity attack [33], we obtain $\mathrm{Pr}\left[E\right]\geq \epsilon \left(1-\frac{1}{q}\right)\frac{1}{q_{H_{1}}q_{H_{3}}}$.

Define $E_{0}=AskH\vee Derr$. There is no greater over $\frac{1}{2^{\lambda }}$ advantage that $A_{4}$ will forge a valid $\left(\sigma_{i^{*}},\rho_{j^{*}},C^{*}\right)$ when $E_{0}$ does not happen because $O_{H}$, $O_{H_{4}}$, and $O_{H_{6}}$ are random oracles. Hence, $\mathrm{Pr}\left[E|\neg E_{0}\right]=\frac{1}{2^{\lambda }}$. We obtain

$$\begin{array}{cc} \mathrm{Pr}[E] & \leq \mathrm{Pr}\left[E|\neg E_{0}\right]\mathrm{Pr}\left[\neg E_{0}\right]+\mathrm{Pr}\left[E_{0}\right]\leq \frac{1}{2^{\lambda }}\left(1-\mathrm{Pr}\left[E_{0}\right]\right)+\mathrm{Pr}\left[E_{0}\right]=\frac{1}{2^{\lambda }}+\left(1-\frac{1}{2^{\lambda }}\right)\mathrm{Pr}\left[E_{0}\right]\leq \frac{1}{2^{\lambda }}+\mathrm{Pr}\left[E_{0}\right]. \end{array}$$

Therefore, we obtain

$$\begin{array}{cc} \mathrm{Pr}[E_{0}] & =\mathrm{Pr}\left[AskH\vee Derr\right]=\mathrm{Pr}\left[AskH\right]+\mathrm{Pr}\left[Derr\right]\geq \mathrm{Pr}\left[E\right]-\frac{1}{2^{\lambda }}. \end{array}$$

Subsequently, we obtain

$$\begin{array}{cc} \mathrm{Pr}[AskH] & \geq \epsilon \left(1-\frac{1}{q}\right)\frac{1}{q_{H_{1}}q_{H_{3}}}-\frac{1}{2^{\lambda }}-\mathrm{Pr}\left[Derr\right]\geq \epsilon \left(1-\frac{1}{q}\right)\frac{1}{q_{H_{1}}q_{H_{3}}}-\frac{1+q_{D}}{2^{\lambda }}. \end{array}$$

Obviously, when AskH occurs, the Gap-BDH assumption can certainly be addressed by B. B addresses the Gap-BDH assumption with advantage $\epsilon^{\prime }=\mathrm{Pr}\left[B\;success\right]=\mathrm{Pr}\left[AskH\right]\geq \epsilon \left(1-\frac{1}{q}\right)\frac{1}{q_{H_{1}}q_{H_{3}}}-\frac{1+q_{D}}{2^{\lambda }}.$

## 6. Performance Evaluation

We first give the functionality and security comparisons, then give the comparisons of the computational overhead and communication overhead.

In Table 1, we compare our proposed IBME-ET with the related schemes (i.e., IB-ME [22], IBEET [3,15], and IBSC-ET [7]) in terms of functionality and security. It can be seen that the IB-ME scheme in [22] ensures the confidentiality, authenticity, and anonymity of data stored in the cloud, but does not achieve CCA security nor provide equality test functionality without losing the confidentiality, authenticity, and anonymity of the data. The IBEET schemes in [3,15] ensure the confidentiality of the data, but neither offer the authenticity and anonymity of data, nor provide equality test functionality without losing the confidentiality, authenticity, and anonymity of the data. Moreover, although the scheme in [3] was the first proposed IBEET scheme, it fails to achieve CCA security. Hence, the IBEET scheme that achieves CCA security was proposed in [15]. The IBSC-ET scheme in [7] ensures the confidentiality and authenticity the data and achieves CCA security, but neither ensures the anonymity of data, nor provides the equality test functionality without losing the confidentiality, authenticity, and anonymity of the data. As a result, only our proposed IBME-ET can realize all the functionality and security, which not only ensures the confidentiality, authenticity, and anonymity of the data stored in the cloud and achieves CCA security, but also provides equality test functionality for ciphertexts generated under different identities without losing the confidentiality, authenticity, and anonymity of the data.

Note that the IB-ME scheme in [22] implements only CPA security. This means that the ciphertexts are malleable. When a valid plaintext/ciphertext pair of the sender and receiver is given, an attacker can utilize it to fake a valid ciphertext of any message, in this way to break the authenticity of the ciphertext stored in the cloud. Moreover, the IB-ME scheme in [22] cannot provide equality test functionality for ciphertexts. Obviously, the IB-ME scheme in [22] is not applicable to cloud storage application scenarios. In addition, it was proven in [15] that the computational overhead and communication overhead of the IBEET scheme in [15] are comparable to those of the IBEET scheme in [3]; however, the IBEET scheme in [15] achieves stricter CCA security while the IBEET scheme in [3] only achieves CPA security. Therefore, we only compared our proposed IBME-ET with the most-related schemes (i.e., IBEET [15] and IBSC-ET [7]) in terms of computational overhead and communication overhead.

Table 2 shows the computational overhead comparison, which theoretically analyzes the computational cost of our proposed scheme and the comparative schemes with regard to encryption key generation (indicated as SKGen ), decryption key generation (indicated as RKGen), encryption (indicated as Enc), decryption (indicated as Dec), authorization (indicated as Auth), and the equality test (indicated as Test). For the analysis, we concentrated on the operations that consumed the most time, including hash-to-point, bilinear pairing, and exponentiation. Notably, the authorization algorithms of the schemes in [7,15] have no computational cost. This is because both schemes directly use the partial decryption private key as the trapdoor regardless of anonymity. The communication overhead comparison is given in Table 3, which theoretically analyzes the communication cost of our proposed scheme and the comparative schemes with regard to the encryption private key, decryption private key, trapdoor, and ciphertext.

**Figure 3:**
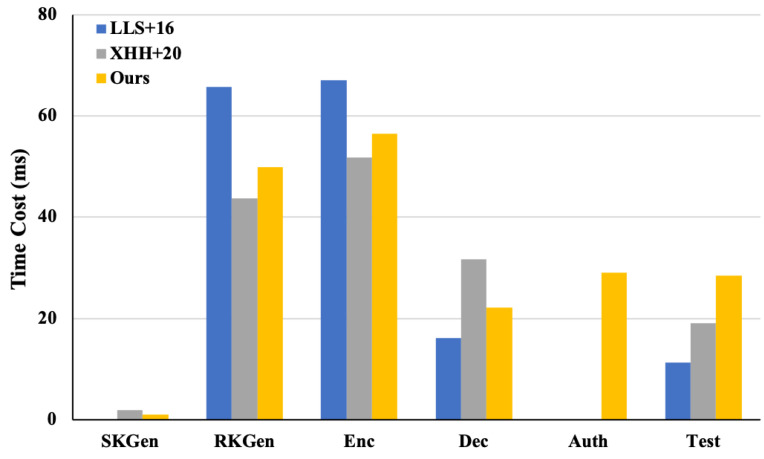
Computational overhead comparison with LLS+16 [15] and XHH+20 [7].

**Figure 4:**
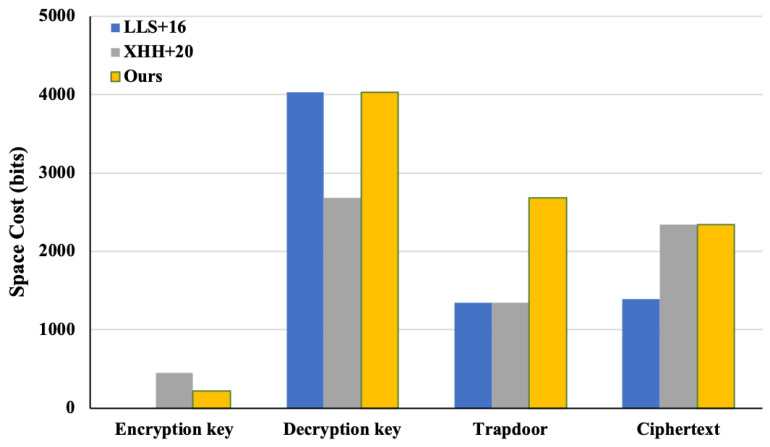
Communication overhead comparison with LLS+16 [15] and XHH+20 [7].

In order to compare the computational and communication overhead of our proposed scheme with the comparative schemes more intuitively, we used Charm 0.50 in Python 3.6.9 to implement these schemes. The experimental environment was configured as follows: Intel(R) Xeon(R) Platinum 8124M CPU @ 2.70 GHz (Intel Corporation, Santa Clara, CA, USA), 16 GB memory, and Ubuntu 18.03 LTS. The experiments were instantiated using the MNT224 curve in Charm and employed the Python module timeit for the time measurements. Figure 3 shows the experimental computational overheads of these schemes, and Figure 4 shows the experimental communication overheads of these schemes.

From Table 1, Table 2 and Table 3 and Figure 3 and Figure 4, we can conclude that, with a small sacrifice in computational and communication efficiency, our IBME-ET scheme not only offers the confidentiality, authenticity, and anonymity of the data and achieves CCA security, but also provides equality test functionality for ciphertexts generated under different identities without losing the confidentiality, authenticity, and anonymity of the data. Other related schemes cannot support this feature.

## 7. Conclusions

In this paper, we presented the primitive of the IBME-ET, which not only offers the confidentiality, authenticity, and anonymity of data and achieves CCA security, but also provides equality test functionality for ciphertexts generated under different identities without losing the confidentiality, authenticity, and anonymity of the data. More precisely, we introduced the system model and definition of the IBME-ET. With respect to the confidentiality, authenticity, and anonymity, we formalized the security models for the IBME-ET. Finally, we proposed a concrete IBME-ET scheme, and our scheme was confirmed to be secure and practical by proving its security and evaluating its performance.

## Figures and Tables

**Table 1:** Comparison of functionality and security.

	Equality Test	Confidentiality	Authenticity	Anonymity	
				Sender	Receiver
[22]	**✗**	CPA	**✓**	**✓**	**✓**
[3]	**✓**	CPA	**✗**	**✗**	**✗**
[15]	**✓**	CCA	**✗**	**✗**	**✗**
[7]	**✓**	CCA	**✓**	**✗**	**✗**
Ours	**✓**	CCA	**✓**	**✓**	**✓**

**Table 2:** Comparison of computational overhead.

	SKGen	RKGen	Enc	Dec	Auth	Test
[15]	-	$3\hat{h}+3\hat{e}$	$3\hat{h}+3p+6e$	3p+2e	0	2p+2e
[7]	2h+2e	$2\hat{h}+2\hat{e}$	$2\hat{h}+2p+5e+\hat{e}$	$2h+5p+2e+\hat{e}$	0	4p
Ours	h+e	$2\hat{h}+3\hat{e}$	$2\hat{h}+3p+5e+\hat{e}$	$h+3p+2e+\hat{e}$	$h+p+4\hat{e}$	6p

$e,\hat{e}$ are exponentiation operations in G and $\hat{G}$, respectively. $h,\hat{h}$ are hash-to-point operations in G and $\hat{G}$, respectively. *p* is the pairing operation.

**Table 3:** Comparison of communication overhead.

	Encryption Key	Decryption Key	Trapdoor	Ciphertext
[15]	-	$3|\hat{G}|$	$|\hat{G}|$	4|G|+5λ
[7]	2|G|	$2|\hat{G}|$	$|\hat{G}|$	$3|G|+|\hat{G}\left|+\right|Z_{q}|+\lambda$
Ours	|G|	$3|\hat{G}|$	$2|\hat{G}|$	$3|G|+|\hat{G}\left|+\right|Z_{q}|+\lambda$

$\left|G|,\right|\hat{G}|$ are the sizes of the elements in groups G and $\hat{G}$, respectively. $|Z_{q}|$ is the size of the elements in $Z_{q}$, and λ is the security level.

## Data Availability

Data are contained within the article.
